# Admixture in a butterfly species complex creates a genomic mosaic of ancestry with distinct histories for different chromosomes

**DOI:** 10.1101/2025.11.28.691233

**Published:** 2025-11-29

**Authors:** Zachariah Gompert, Lauren K. Lucas, Alia Donley, Matt L. Forister, James A. Fordyce, Chris C. Nice

**Affiliations:** 1 Department of Biology, Utah State University, Logan, UT, 84322, USA; 2 Ecology Center, Utah State University, Logan, UT, 84322, USA; 3 Department of Biology, University of Nevada, Reno, NV, 89557, USA; 4 Department of Ecology & Evolutionary Biology, University of Tennessee, Knoxville, TN, 37996 USA; 5 Department of Biology, Texas State University, San Marcos, TX, 78666, USA

**Keywords:** admixture, hybridization, sex chromosomes, species, population genomics, Lepidoptera

## Abstract

Hybridization and admixture give rise to populations with mosaic genomes composed of ancestry segments with distinct origins and histories. Despite a growing number of documented cases of admixture in nature, relatively little is known about how mosaic patterns of ancestry vary simultaneously across the genome and among multiple populations or lineages of hybridizing species. Here, using pooled whole-genome sequence data from more than 20 populations representing three nominal species and multiple admixed or taxonomically ambiguous populations, we show that widespread admixture in *Lycaeides* butterflies has resulted in distinct evolutionary histories within and among chromosomes, with the most notable differences between the autosomes and the Z sex chromosome. Many populations exhibit substantial evidence of mixed ancestry on the autosomes, whereas the Z chromosome shows a more tree-like evolutionary history. In some cases, the predominant ancestry of the autosomes and the Z chromosome differ. We also find evidence of variation in ancestry within chromosomes, though this appears more idiosyncratic. We also show that differences in autosomal versus Z-chromosome ancestry have the potential to shape ecologically important trait variation. Specifically, using genome-wide association mapping, we demonstrate that wing-pattern elements–traits known to influence mate preference–are affected by genetic variants on both the autosomes and the Z chromosome. In sum, our results show that different chromosomes can exhibit distinct evolutionary histories, with the Z chromosome suggesting more discrete evolutionary lineages or species than the autosomes. These findings have implications both for our basic understanding of species and species boundaries and for how we view the sources of genetic and phenotypic variation that fuel ongoing evolutionary change.

## Introduction

The role of hybridization in adaptation and speciation has been the subject of long-standing debate, but growing evidence supports its common occurrence and (sometimes) creative role in evolution (Mallet, 2005; Taylor & Larson, 2019; Edelman & Mallet, 2021; Peñalba et al., 2024). Recent studies have shown that the genomes of many organisms–including fish (Meier et al., 2023; Langdon et al., 2024), butterflies (Rosser et al., 2024; Boman et al., 2025), flies (Suvorov et al., 2022), snakes (Nikolakis et al., 2022), sunflowers (Hübner et al., 2019), conifers (Bolte et al., 2024) and humans (Iasi et al., 2024; Li et al., 2024)–contain DNA segments that were inherited from different species through admixture and introgressive hybridization. We also now know that introgression varies in extent across the genome. For example, introgression is sometimes limited to a few regions that drive adaptation (e.g., Oziolor et al., 2019; Rossi et al., 2024). In other cases introgression is genomically widespread, excluding only a few regions of the genome that negatively affect hybrid fitness (e.g., Poelstra et al., 2014; Schield et al., 2024).

The genomic extent of mixed ancestry and introgression is relevant for our understanding of reproductive isolation and the nature of species. Whereas foundational studies of speciation emphasized mechanisms of reproductive isolation and considered it a property of whole organisms or species (e.g., Dobzhansky, 1937; Mayr, 1963; Coyne & Orr, 1989), genomic studies typically measure reproductive isolation as a reduction in effective gene flow and thus regard it as something that varies across the genome (Barton & Bengtsson, 1986; Wu, 2001; Westram et al., 2022). Under this genetic framework, genomic regions with limited introgression can function as good species, that is, distinct genetic clusters or independently evolving lineages, even if admixture and introgression are widespread across much of the genome. Consequently, understanding how patterns of introgression and admixture vary across the genome is important for understanding the nature of species and the structure of biological diversity.

Along these lines, patterns of ancestry and introgression often differ between autosomes and sex chromosomes. For example, numerous hybrid zone studies have shown reduced introgression at Z or X loci relative to autosomal loci (Dod et al., 1993; Carling & Brumfield, 2008; Storchová et al., 2010; Maroja et al., 2015; Gompert et al., 2017; Hooper et al., 2019; Chaturvedi et al., 2020; Banker et al., 2022; Swaegers et al., 2022; Zhang et al., 2023). Such patterns, together with findings from interspecific crosses, trait mapping, transcriptomics, population genomics, and molecular evolution, suggest that sex chromosomes play a disproportionate role in speciation and the maintenance of species boundaries (Sperling, 1994; Masly & Presgraves, 2007; Kitano et al., 2009; Irwin, 2018; Payseur et al., 2018; Sánchez-Ramírez et al., 2021). This is consistent with the “large X-effect” and Haldane’s rule, and may arise because of faster sex chromosome evolution or because sex-linked incompatibilities are expressed in the heterogametic sex (Orr, 1997; Carling & Brumfield, 2008; Presgraves, 2008; Lenormand & Roze, 2025). Regardless of the mechanism, and particularly from the perspective of a genetic conception of reproductive isolation, the nature of species and the organization of biodiversity may thus generally differ between sex chromosomes and autosomes, with sex chromosomes behaving more like well-defined species with a bifurcating, tree-like history. The same could be true for other non-recombining regions of the genome, such as some chromosomal rearrangements.

Many studies have examined patterns of admixture and introgression in nature, but much of this work has focused either on introgression between individual species pairs or has not explicitly analyzed how introgression varies among and within chromosomes (but see, e.g., Thawornwattana et al., 2023; Langdon et al., 2024; van der Heijden et al., 2025). Thus, additional studies that quantify patterns of mixed ancestry and introgression across multiple populations and species, and that assess how such patterns differ across the genome, are needed to better understand genome-wide variation in evolutionary histories and entities. Moreover, combining such work with trait genetics is important for assessing the contribution of hybridization to fueling phenotypic adaptation (Marques et al., 2019).

Here, we use North American butterflies in the genus *Lycaeides* to address this gap (*Lycaeides* has been reclassified as being within the genus *Plebejus* but we retain the older name name for consistency with past work and because it more precisely defines our focal lineages; Talavera et al., 2013). In North America, *Lycaeides* comprise a complex of several nominal species (i.e., *L. anna*, *L. idas*, *L. melissa*, and *L. samuelis*) as well as admixed or taxonomically ambiguous lineages (e.g., the Sierra Nevada, Warner Mountain, and Rocky Mountain populations described below) (Nabokov, 1949; Gompert et al., 2006, 2010; Forister et al., 2011; Gompert et al., 2012; Nice et al., 2013; Gompert et al., 2014). At least one contemporary hybrid zone also exists (Chaturvedi et al., 2020; Zhang et al., 2023). Despite past and ongoing hybridization, *Lycaeides* species differ in host plant use and preference, voltinism and phenology, male genitalic morphology, wing patterns, and mate preference (Nice & Shapiro, 1999; Fordyce et al., 2002; Nice et al., 2002; Forister et al., 2006; Lucas et al., 2008; Forister et al., 2009; Gompert et al., 2013b,a; Lucas et al., 2018). Putative admixed populations display unique combinations of parental, intermediate and transgressive traits and ecologies. For example, putative admixed lineages in the Sierra Nevada and White Mountains (hereafter collectively referred to as Sierra Nevada populations) occupy alpine habitats, feed on an alpine endemic host plant, *Astragalus whitneyi*, and have a unique, locally adaptive lack of egg adhesion (Gompert et al., 2006; Nice et al., 2013). Suspected admixed populations in the Warner Mountains also use an *Astragalus* host and exhibit a mosaic of *L. anna*, *L. melissa*, and intermediate morphological traits (Nice et al., 2013). Admixed populations are also known from the Rocky Mountains (specifically in and around Jackson Hole, hereafter referred to as Rocky Mountain populations); these mostly use the same host and occupy similar habitats (montane forest meadows and sagebrush-dominated buttes) as nearby *L. idas*, but have *L. melissa*-like genitalic morphology and show evidence of mixed *L. melissa* and *L. idas* ancestry (Gompert et al., 2010, 2012, 2013b).

Past population genomic studies of *Lycaeides* suggest that genetic differentiation and introgression vary across the genome, but these inferences have been based either on limited genomic sampling (e.g., genotyping-by-sequencing [GBS] data), limited spatial sampling (e.g., a single hybrid zone), or both (Gompert et al., 2012; Chaturvedi et al., 2020; Zhang et al., 2023). Moreover, previous work has largely lacked *Lycaeides* samples from Alaska (where colonization of North America occurred) and outgroup taxa from Eurasia (where the closest extant relatives of the North American taxa occur) (Vila et al., 2011a; Toro-Delgado et al., 2025), which together have limited our ability to infer the direction of evolutionary change. In the current study, we overcome these limitations by using pooled whole-genome sequencing of 23 *Lycaeides* populations–comprising the nominal species *L. anna*, *L. idas* (including Alaskan individuals), and *L. melissa*, as well as several named and unnamed putative hybrid taxa–combined with population genomic and phylogenetic methods to quantify patterns of ancestry and admixture throughout this genus and within and among chromosomes. We address two main questions: (i) how much do patterns of ancestry vary across the genome, and (ii) does the Z chromosome exhibit a more tree-like evolutionary history than the rest of the genome? Our central hypothesis is that the Z chromosome has a disproportionate effect on ecologically important traits and hybrid fitness, and thus exhibits a tree-like history despite widespread hybridization and introgression across much of the genome. Alternatively, admixture could be quite limited overall, or prevalent and not restricted to the autosomes. Finally, we combine our core investigation with genome-wide association mapping of previously published wing pattern data. This allows us to test whether the detected admixture has biologically meaningful phenotypic consequences, and whether the Z chromosome contributes disproportionately to variation in wing pattern, which influences mate recognition (Fordyce et al., 2002; Gompert et al., 2013b), as predicted by our central hypothesis.

## Methods

### Genetic data

**Figure 1:**
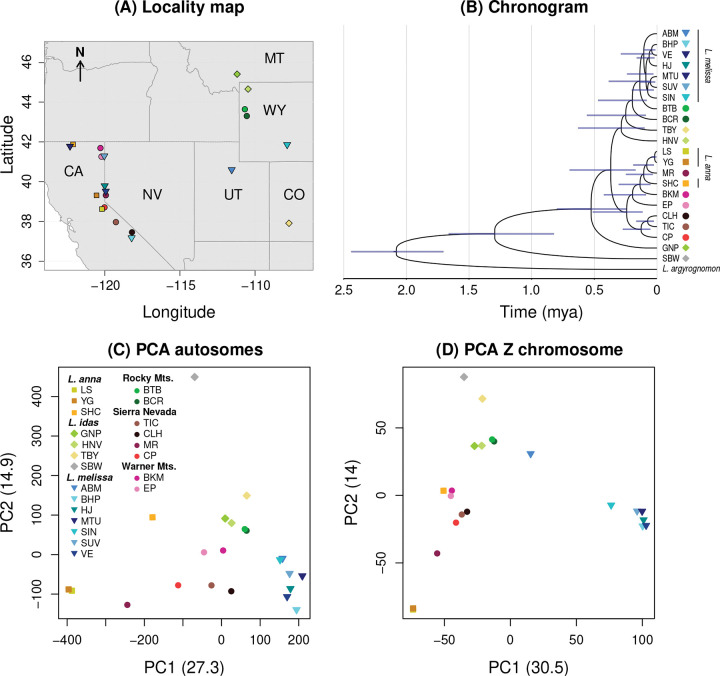
Sample localities and summary of phylogenetic relationships and genetic variation. (A) The map shows the locations of the butterfly populations in the contiguous, western USA (the *L. idas* population from Alaska, SBW, and the outgroup, *L. argyrognomon*, are not shown). State abbreviations are given. (B) A chronogram depicts our Bayesian reconstruction of the (consensus) best-tree approximation to the evolutionary history of *Lycaeides*. Time estimates are in millions of years ago (mya) and shaded bars denote 95% highest-posterior densities for node ages. Statistical summaries of patterns of population genetic structure for the sampled *Lycaeides* based on principal component analyses of allele frequencies for the autosomes (C) or Z chromosome (D). Colored symbols denote populations, which are organized in the inset legend by species or geographic region for taxonomically ambiguous populations.

We extracted DNA from 996 butterflies from 23 populations, including 142 *L. anna* (three populations), 148 *L. idas* (four populations), 339 *L. melissa* (seven populations), 357 unnamed, admixed or taxonomically ambiguous *Lycaeides* (eight populations) and 10 *L. argyrognomon* (one population) as an outgroup, using Qiagen’s DNeasy 96 Blood and Tissue Kit (Table 1 and Figure 1A). We measured DNA concentrations with a flourescence microplate reader and combined equal total amounts of DNA (by mass) from butterflies for each population. These population pools of DNA were used for pooled whole-genome sequencing. Sequencing libraries were constructed and sequenced on the DNBseq platform (paired-end 150 bp reads) by BGI. We repeated the pooling, library preparation and sequencing twice for four populations for quality control. Raw sequences were trimmed and filtered with SOAPnuke (Chen et al., 2018). A detailed description of library preparation, sequencing and read filtering is provided in the Online Supplemental Material (OSM).

We aligned the sequence reads to the *L. melissa* genome using bwa-mem2 (version 2.0pre2) (Li & Durbin, 2009; Vasimuddin et al., 2019). PCR duplicates were then marked and removed with samtools (version 1.16) (Li et al., 2009; Ebbert et al., 2016). We called genetic variants using bcftools (version 1.16) (Li, 2011) and filtered the resulting initial SNP set with GATK (version 4.1.4.1) (McKenna et al., 2010), resulting in 15,914,826 SNPs. We extracted the counts of high-quality reads supporting the reference and alternate allele for each SNP and population; we then used these counts to estimate allele frequencies and conduct downstream population genomic and phylogenetic analyses. See the OSM for additional details about alignment, variant calling, SNP filtering and allele frequency estimation.

### Phylogeny and population structure

We began by characterizing the overall relationships among the *Lycaeides* populations. We took two complementary approaches. First, we used Beast (version 2.7.7) to estimate a time-calibrated phylogeny (a chronogram) from a subset of genome-wide SNPs. Specifically, we used a concatenated set of 5408 SNPs interspersed across the genome (as required for computational feasibility); variable sites within populations were coded as the more common allele when its frequency was ≥ 0.95 and as ambiguous (N) when this was not true. Invariant sites were also accounted for in the model. We used existing estimates for the divergence of *L. argyrognomon* and North American *Lycaeides* and for the colonization of North America by *Lycaeides* to construct informative priors for the molecular clock in our analysis (Vila et al., 2011b; Kawahara et al., 2023). See the OSM for a detailed description of the phylogenetic model and priors used. Second, we summarized patterns of genetic variation and genetic similarity using a principal components analysis (PCA) of genome-wide allele frequencies. We did this in R. We separately analyzed the 14,907,399 autosomal SNPs and the 1,007,427 Z chromosome SNPs. In both cases, we included only the *Lycaeides* populations (i.e., we excluded the outgroup, *L. argyrognomon*) and analyzed the centered, but not standardized, allele frequency matrix.

### Tests for tree-like histories

We next asked how well the evolutionary relationships among the *Lycaeides* populations could be explained by a bifurcating tree for each of the 22 autosomes and the Z chromosome. To this end, we first used TreeMix (version 1.13) to estimate a composite maximum-likelihood population tree for each chromosome from the population allele-frequency covariance matrix. We rooted each tree using *L. argyrognomon* and used a block-resampling approach with blocks of 100 SNPs to estimate the unobserved population allele-frequency covariance matrix from the observed sample covariance matrix. We then added migration (admixture) edges to the maximum-likelihood tree for each chromosome to generate admixture graphs. This procedure was repeated 20 times for each chromosome and number of migration edges to increase the likelihood of convergence on an optimal solution. We calculated the proportion of the allele-frequency covariance for each chromosome explained by each graph, that is, for graphs with zero (a bifurcating tree) to eight migration edges. A higher proportion of covariance explained with fewer migration edges indicates a more tree-like evolutionary history for that chromosome.

We then tested for a relationship between chromosome size (in base pairs) and the proportion of covariance explained by trees and graphs with a given number of migration edges. Assuming recombination per base pair is lower on longer chromosomes (i.e., that recombination per chromosome is approximately constant) and that selection against deleterious foreign alleles removes larger DNA blocks on longer chromosomes (e.g., Brandvain et al., 2014; Schumer et al., 2018), we might expect allele-frequency covariances to be better explained by a bifurcating tree (or a graph with only a few admixture events) for short chromosomes than for long chromosomes. In contrast, we might expect no such relationship with chromosome size for admixture graphs with many migration events. We fit Bayesian regression models to test for these patterns, as described in the OSM. Finally, we examined the nature and weights–proxies for admixture proportions–of the migration edges across chromosomes and graphs to identify the most noteworthy instances of putative admixture.

### Genomic variation in evolutionary relationships

We used the program CASTER (version 1.20.2.5) to quantify variation in tree topologies within and among chromosomes and thereby further assess genome-wide variability in evolutionary histories (Zhang et al., 2025). CASTER implements a site-based, coalescent-aware species-tree estimation method for rapid, genome-scale phylogenetic analyses. Importantly for our purposes, the approach produces a score for each SNP that can be used to detect changes in evolutionary histories across the genome.

We first used CASTER to estimate trees for each chromosome. We used the CASTER-site model for this, set *L. argyrognomon* as the outgroup, and coded SNPs within populations with the common allele when its frequency exceeded 0.95 and otherwise as ambiguous (N). We used the generalized Robinson-Fould distance to quantify differences in tree topologies among chromosomes; this metric captures differences in mutual clustering information among tree topologies (Smith, 2020a). We did this using the TreeDist R package (version 2.11.0) (Smith, 2020b). We then asked whether the tree distances among chromosomes were greater than expected under a null model where all chromosomes shared the same species tree (i.e., under a null model where SNPs were permuted among chromosomes). SNPs were repeatedly randomized among chromosomes (*n* = 50 permutations) to generate null expectations.

We next used CASTER to quantify variability in evolutionary histories both within and among chromosomes. To do this, we computed scores for alternative four-taxon trees in sliding windows along chromosomes (similar to earlier methods for topology weighting, e.g., Martin & Van Belleghem, 2017). We focused on four-taxon sets representing putative cases of admixture suggested by our TreeMix results, differences in tree topologies among chromosomes from CASTER, and results from previous population genomic studies of *Lycaeides* (Gompert et al., 2006, 2010; Nice et al., 2013; Gompert et al., 2014), as well as control four-taxon sets where admixture was not expected. In all cases, the Alaskan *L. idas* population (SBW) was included as an outgroup for the other three populations analyzed. We computed scores in 10-kilobase windows using CASTER-slidingwindow (https://github.com/chaoszhang/ASTER) and then averaged and normalized these scores across 50 kilobase sliding windows in R. The normalized scores sum to one over the possible topologies. Substantial variation in scores within and among chromosomes, especially over large genomic regions, is strongly suggestive of admixture, with the scores representing the relative support for, or contribution of, different histories (Zhang et al., 2025).

### Genetic mapping of wing pattern

Finally, to go beyond genomic patterns and gain some initial insights into the relevance of admixture for phenotypic evolution, we last asked whether differential evolutionary histories and ancestry for autosomes versus the Z chromosome (as documented in the Results) would be expected to have meaningful phenotypic consequences for ecologically important traits in admixed populations. We used wing pattern as a test case for this, and specifically asked whether both autosomes and the Z sex chromosome contributed substantially to wing pattern variation. *Lycaeides* wing patterns comprise a series of black spots and aurorae (border ocelli with black and orange pigments, as well as structural colors) (Nabokov, 1943; Fordyce et al., 2002; Lucas et al., 2018). We re-analyzed wing pattern measurements (the sizes of 17 wing pattern elements) and corresponding genetic data from three *Lycaeides* populations: YG (*L. anna*, *n* = 100), GNP (*L. idas*, *n* = 98), and SIN (*L. melissa*, *n* = 97) (Lucas et al., 2018). We used a polygenic genome-wide association mapping approach to estimate the proportion of the additive genetic variance explained for each trait by each chromosome in each of the three populations (Zhou et al., 2013). This was done by fitting Bayesian sparse linear mixed models in gemma (version 0.95a) (Zhou & Stephens, 2012). We ran 20 chains of 1,000,000 iterations (500,000 iteration burnins) for each trait and population, and estimated additive genetic variances from the model-averaged SNP effect estimates and population allele frequencies. Details of these data, DNA sequence alignment and variant calling, and genetic mapping are provided in the OSM.

## Results

### Phylogeny and population structure

Bayesian phylogenetic analysis suggests that the sampled North American *Lycaeides* diverged from a common ancestor approximately 1.3 million years ago (mya) (95% highest posterior interval [HPI] = 0.8–1.7), with the most basal split separating the Alaskan *L. idas* population (SBW) from the other populations, which occupy the contiguous United States and diverged from each other around 0.5 mya (95% HPI = 0.2–0.8) (Figure 1A,B). Our results indicate that *L. idas* is paraphyletic, even when considering only populations from the contiguous United States, whereas *L. melissa* and *L. anna* are monophyletic, at least if putative admixed populations (specifically MR) are excluded. The most recent common ancestors of *L. melissa* and *L. anna* were estimated to have existed 130 thousand (95% HPI = 29–238) and 120 thousand (95% HPI = 33–247) years ago, respectively. The taxonomically ambiguous and putative hybrid lineages from the Rocky Mountains, Sierra Nevada, and Warner Mountains generally formed paraphyletic clades nested within *L. idas*, but not within *L. melissa* or *L. anna* (with the exception of MR). Results from PCA were largely consistent with the phylogenetic tree but also highlighted differences in population genetic structure between the autosomes and the Z chromosome (Figure 1C,D). In both cases, PC1 separated *L. anna* and *L. melissa*, with *L. idas* and taxonomically ambiguous populations showing intermediate PC1 scores; however, these populations tended to be more similar to *L. anna* (i.e., lower PC1 scores) for the Z chromosome than for the autosomes. Interestingly, one *L. melissa* population (ABM) exhibited a more *L. anna*-like PC1 score for the Z chromosome. PC2 separated the Alaskan *L. idas* population (SBW) from the other *Lycaeides* populations, with a notable gap in PC space for the autosomes but not for the Z chromosome, which instead showed a more continuous distribution along PC2.

### Tests for tree-like histories

**Figure 2:**
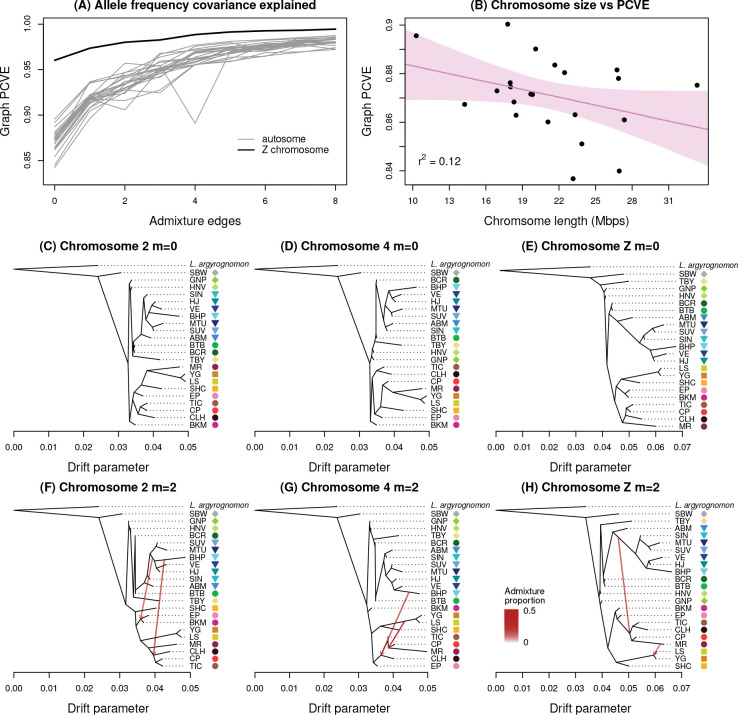
Patterns of genetic variation explained by population trees and admixture graphs from TreeMix. Panel (A) shows the proportion of allele frequency covariance explained (PCVE) by graphs with 0 to 8 migration edges (a graph with 0 edges is a bifurcating tree). Gray lines correspond with the 22 autosomes; the black line denotes the Z chromosome. Panel (B) depicts the PCVE for a bifurcating tree (0 migration edges) for each of the 22 autosomes, which are denoted by black points. The solid line and shaded region show the best fit line and 95% equal-tail probability intervals (ETPIs) from a Bayesian regression model (*β* = −0.41, 95% ETPI = −0.90–0.11, post. prob. *β* < 0 = 0.937, least squares regression *r*^2^ = 0.12). Panels (C), (D) and (E) depict population graphs for chromosomes 2, 4 and Z with no migration events, with units in terms of a drift parameter proportional to evolutionary change. Colored symbols and population IDs correspond with Table 1 and Figure 1. The *L. argyrognomon* population is included as an outgroup. Panels (F), (G) and (H) show graphs for the same three chromosomes with m = 2 migration edges (red arrows). The admixture proportions associated with each are indicated by the intensity of each red arrow, but in this case all migration edges shown had high weights: 0.30–0.45. See Figures S2–S24 for the full set of graphs for all chromosomes and 0 to 6 migration edges.

A strictly bifurcating tree (i.e., a population graph with 0 migration edges) explained patterns of allele frequency covariance better for the Z chromosome (96.0%) than for the autosomes (mean = 87.1%, minimum = 84.2%, maximum = 89.6%) (Figure 2A). Adding migration edges increased the variance explained for all chromosomes, but especially for the autosomes, such that with eight migration edges the graph explained 99.5% of the allele frequency covariance for the Z chromosome and an average of 98.1% for the autosomes (range = 97.2–98.7%). We found modest evidence for a moderate negative relationship between the proportion of allele frequency covariance explained by a bifurcating tree and chromosome size for autosomes (*β* = −0.41, 95% ETPI = −0.90–0.11, post. prob. *β* < 0 = 0.937, least squares regression *r*^2^ = 0.12) (Figures 2B and S1). Thus, smaller autosomal chromosomes have more tree-like evolutionary histories, though not strongly so. A similar pattern was observed for admixture graphs with few migration edges, but the relationship reversed for graphs with four or more migration edges, such that a greater proportion of the allele frequency covariance was explained by these graphs for larger chromosomes than for smaller ones. Consequently, there was a significant positive correlation between the number of migration edges and the relationship between chromosome size and the proportion of allele frequency covariance explained (Pearson *r* = 0.80, 95% confidence interval [CI] = 0.29–0.96, *P* = 0.0097) (Figure S1).

We detected several admixture events that were consistent or largely consistent across chromosomes (Figure 2C–H and S2–S24). Here, we focus on events from graphs with one or two migration edges, as adding the first few edges produced the largest improvements in the proportion of allele frequency covariance explained and yielded the most easily interpretable results. For example, for autosomes with one migration edge, we found consistent evidence of admixture from *L. melissa* in the history of the Sierra Nevada populations, especially TIC and CLH, and sometimes CP. Based on the tree topology, these populations were often otherwise most closely related to *L. anna* (for chromosomes 21 and 22, the majority ancestry was instead from *L. melissa*, with minority ancestry from the ancestor of MR, CP, and two *L. anna* populations). The inferred *L. melissa* source populations were either BHP or the ancestor of BHP and VE (both from the western USA near the Sierra Nevada). Admixture (migration) weights indicated substantial contributions from this event, with a mean admixture weight or proportion (across chromosomes) of 0.39 (range = 0.27–0.48).

For the case of two migration edges, most chromosomes suggested the same (or similar) admixture event described above, along with evidence of (i) admixture between *L. melissa* and the two Warner Mountains populations (EP and BKM), which were otherwise most closely related to *L. anna* and the Sierra Nevada populations; (ii) admixture in the history of the SHC *L. anna* population; or (iii) admixture within the Sierra Nevada populations (CP, MR, both CP and MR, or all Sierra Nevada populations). These putative admixture events involved introgression from *L. anna*, *L. melissa*, the Sierra Nevada populations, or an ancestor of some subset of these (Figure 2C–H and S2–S24). Here too, the estimated levels of admixture were substantial (mean = 0.37, range = 0.16–0.50). Admixture in the history of MR was also detected for the Z chromosome. For the Z chromosome, we additionally found evidence of early admixture from an ancestral *L. melissa* population in the history of all Sierra Nevada populations (admixture proportions of 0.30 and 0.36). We found little to no evidence of admixture in the history of the Rocky Mountains populations. Patterns of admixture for graphs with three or more migration edges were increasingly complex and explained little additional allele frequency covariance. Nonetheless, even with many migration edges, the estimated migration weights remained considerable, consistent with major contributions of admixture to the genetic compositions and relationships of populations (e.g., for six migration edges, the mean weight was 0.34 for autosomes, with a range of 0.06–0.61).

### Genomic variation in evolutionary relationships

**Figure 3:**
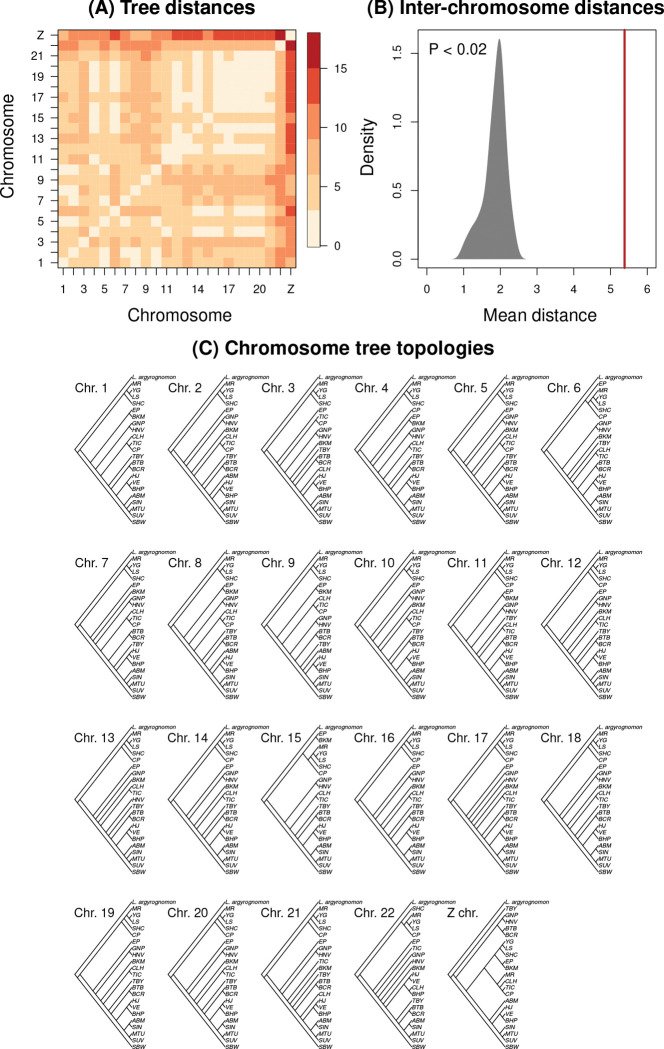
Summary of tree topologies from CASTER. Panel (A) depicts a heatmap showing generalized Robinson-Fould distances between the tree topology for each pair of chromosomes; this metric is based on differences in the mutual clustering information between trees. Panel (B) shows the mean generalized Robinson-Fould distance among trees for all pairs of chromosomes (vertical red line) relative to a null distribution (shaded gray region) generated from randomly assigned SNPs to chromosomes (the observed value exceeds null expectations with *P* < 0.02 (example topologies from one permutation are shown in Figure S26). Panel (C) shows the estimated tree topology (as a cladogram) from the CASTER-site model for each chromosome with *L. argyrognomon* as the outgroup. See Table 1 for population IDs.

We detected 19 unique tree topologies at the chromosome level for the 23 *Lycaeides* chromosomes using CASTER (Figure 3) (we obtained similar results with the CASTER-pair model; Figure S25). Notable differences among chromosomes included the placement of the Sierra Nevada (CP, MR, TIC, and CLH) and Warner Mountains (EP and BKM) populations, as well as the position of GNP and HNV (both *L. idas*) relative to other lineages. The mean Robinson-Foulds distance between pairs of trees was 5.27 (range = 0.00–15.97), with the largest distances observed between the Z chromosome and the autosomes (mean = 11.79, range = 7.13–15.97). The mean tree distance among chromosomes exceeded null expectations, suggesting significant differences in the dominant evolutionary history across chromosomes (average generalized Robinson–Foulds distance among chromosomes, null based on 50 permutations: mean = 1.86, range = 1.01–2.38, *P* < 0.02) (Figures S25B and S26).

**Figure 4:**
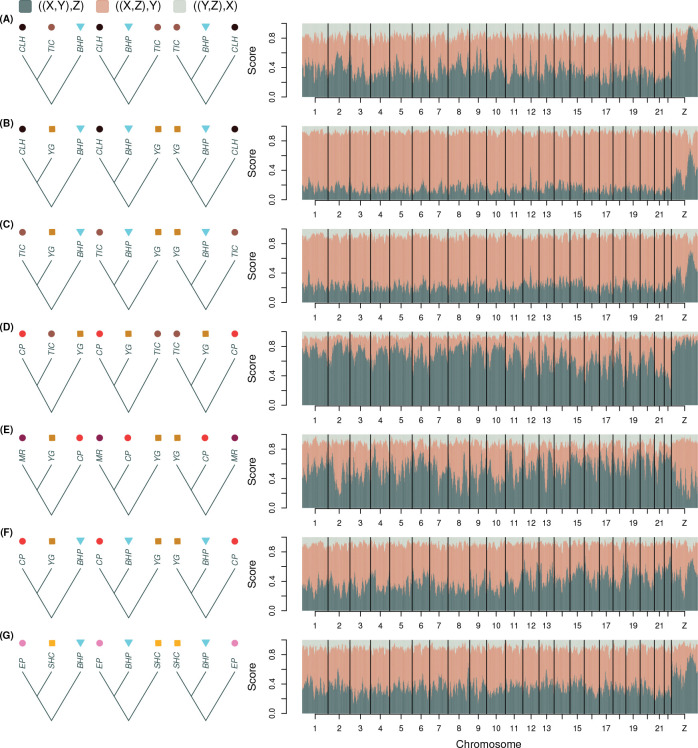
Genomic variation in four-taxon tree topologies for the Sierra Nevada and Warner Mountains populations (SBW, an Alaskan *L. idas* was always used as the outgroup and is not shown). Plots show normalized scores for three tree topologies, which were initially computed in 10 kilobase windows and the averaged over 50 kilobase overlapping sliding windows. Colors denote alternative topologies as shown in the legend, where X, Y and Z denote taxa as depicted to the left of the plots. The ((X,Y),Z) topology was defined to be consistent with the topology of the Bayesian chronogram from Figure 1B. Taxonomic designations for the populations shown in the figure are: *L. anna* = YG and SHC; *L. melissa* = BHP; Sierra Nevada = CP, MR, TIC and CLH; and Warner Mountains = EP.

Patterns of variation in support for alternative four-taxon trees within and among chromosomes provided additional evidence for admixture in the histories of the Sierra Nevada and Warner Mountains populations (Figure 4). Across the seven sets of four-taxon analyses we conducted, mean within-chromosome variance in tree scores (within-chromosome variation) and among-chromosome variance in mean scores (among-chromosome variation) were of similar magnitude (mean within = 0.0055, range = 0.0028–0.0098; mean among = 0.0045, range = 0.0019–0.0083). Below, we highlight several of the most striking patterns from these analyses.

Trees uniting CLH and TIC (two Sierra Nevada populations) with each other, or with *L. anna* (YG) rather than *L. melissa* (BHP), were most strongly supported on the Z chromosome, particularly across two large regions of the chromosome; this was true despite high overall scores for trees uniting CLH (and to a lesser extent TIC) with BHP (Figure 4A–C). Trees uniting CP and TIC (two Sierra Nevada populations) to the exclusion of *L. anna* (YG) were strongly supported overall, especially across the entire Z chromosome and on several of the longer autosomes (Pearson correlation between autosome size and mean score = 0.89, 95% CI = 0.75–0.95, *P* < 0.001; Figure 4D). The MR and CP Sierra Nevada populations likewise formed a clade to the exclusion of *L. anna* (YG) across much of the Z chromosome and on longer autosomes (Pearson correlation = 0.77, 95% CI = 0.52–0.90, *P* < 0.001; Figure 4E). Support for trees where CP and *L. melissa* (BHP) formed a clade exclusive of *L. anna* (YG) also increased with autosome length (Pearson correlation = 0.89, 95% CI = 0.74–0.96, *P* < 0.001) and was higher in the center than at the ends of the Z chromosome, similar to patterns observed for CLH and TIC (Figure 4B,C,F). Distinct patterns of tree support were also observed for the Warner Mountains population (EP) on the Z chromosome, with increased support for EP and *L. anna* (specifically SHC) as a clade exclusive of *L. melissa* across much of the Z chromosome but weaker support near the chromosome center (Figure 4G). Notably, for all seven of these four-taxon analyses, the Z chromosome exhibited distinct tree scores, sometimes supporting the same relationships most strongly supported on the autosomes but to a greater degree (e.g., Figure 4D), and other times supporting an evolutionary history different from that suggested by the autosomes (e.g., Figure 4A–C,G).

**Figure 5:**
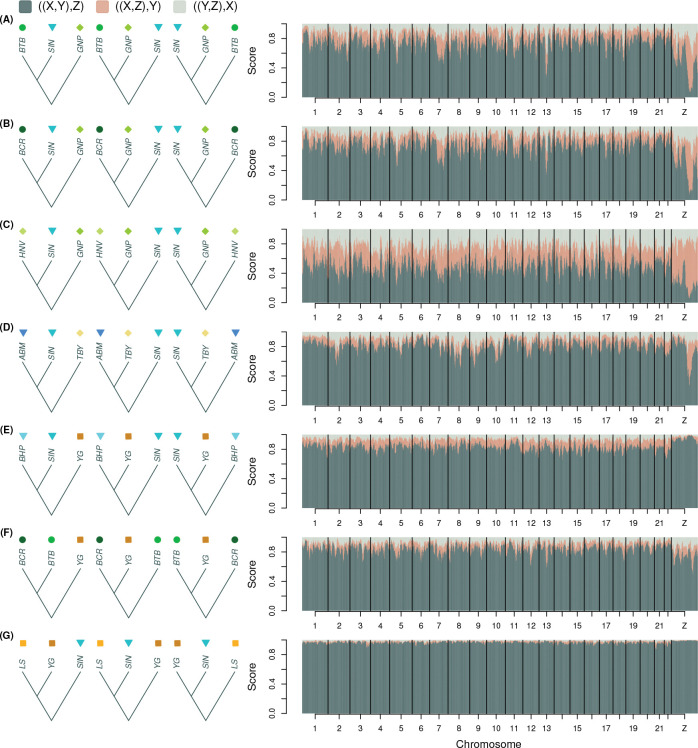
Genomic variation in four-taxon tree topologies for the Rocky Mountains populations and control groups, that is for cases where little or no admixture was expected. SBW, an Alaskan *L. idas* was always used as the outgroup and is not shown. Plots show normalized scores for three tree topologies, which were initially computed in 10 kilobase windows and the averaged over 50 kilobase overlapping sliding windows. Colors denote alternative topologies as shown in the legend, where X, Y and Z denote taxa as depicted to the left of the plots. The ((X,Y),Z) topology was defined to be consistent with the topology of the Bayesian chronogram from Figure 1B. Taxonomic designations for the populations shown in the figure are: *L. anna* = LS and YG; *L. idas* = GNP, HNV and TBY; *L. melissa* = ABM, BHP and SIN; and Rocky Mountains = BCR and BTB.

We also examined relationships within and among chromosomes for the Rocky Mountain populations (BTB and BCR) and nearby *L. melissa* and *L. idas* populations (Figure 5A–D), as well as control four-taxon comparisons where no admixture was suspected (Figure 5E–F). For both Rocky Mountain populations, most of the genome supported recent common ancestry with *L. melissa* (SIN) rather than *L. idas* (GNP) (Figure 5A,B). The main exception was the Z chromosome, which showed strong support for a Rocky Mountain and *L. idas* clade, particularly across a substantial portion of the chromosome; increased support for this clade was also evident on parts of some autosomes (e.g., chromosome 7). Interestingly, the *L. idas* population (HNV) geographically proximate to BTB and BCR also showed moderate to strong support for a closer relationship with *L. melissa* (SIN) than with the other *L. idas* population (GNP), although the majority of the Z chromosome (but not all of it) stood out as a clear exception (Figure 5C). Control cases in which four-taxon trees included either two *L. melissa*, two *L. anna*, or two Rocky Mountain populations along with a more distant relative showed much more consistent support for a single topology across the genome, with generally similar, slightly stronger, or slightly weaker support for this topology on the Z chromosome (Figure 5D–F). The one exception involved the ABM *L. melissa* population; trees uniting this high-elevation *L. melissa* population with a high-elevation *L. idas* population (TBY) rather than with another *L. melissa* population (SIN) received moderate to strong support across a substantial portion of the Z chromosome. Nonetheless, these control cases demonstrate that pronounced variation in support for alternative trees across the genome is not inevitable.

### Genetic mapping of wing pattern

**Figure 6:**
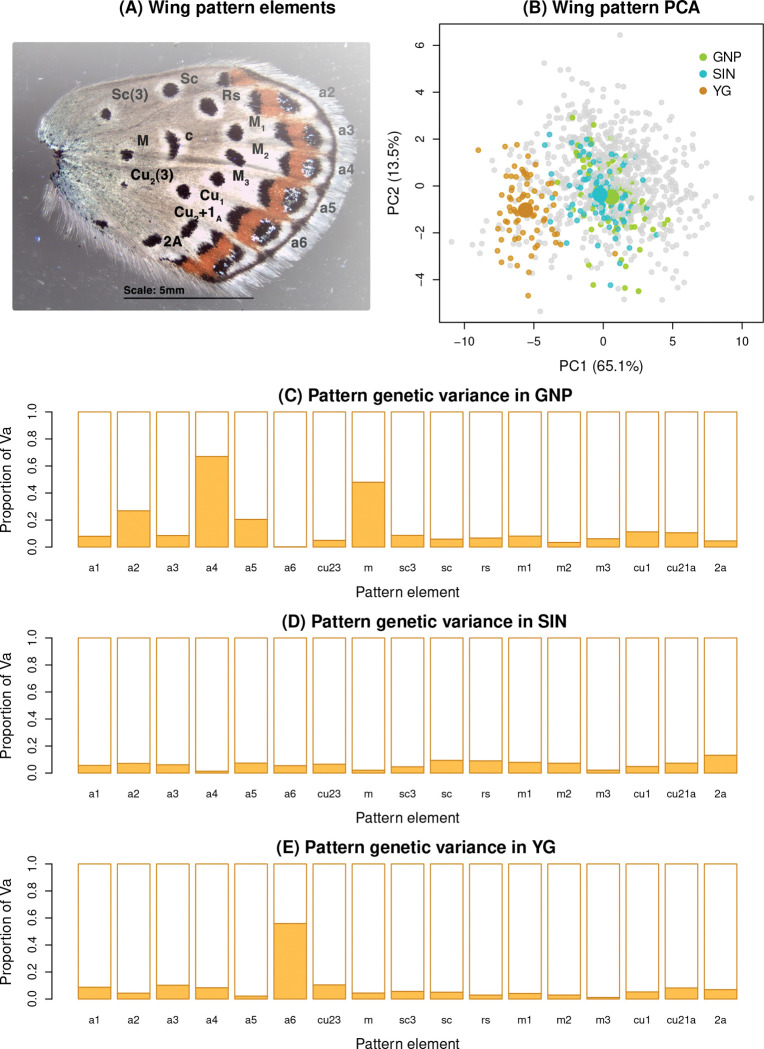
Summary of wing pattern mapping analysis. Panel (A) shows the ventral surface of the hind-wing with the measured black spots and aurorae (a2-a6) labeled. Panel (B) summarized wing pattern variation in this system based on a principal components analysis. PCs 1 and 2 largely capture overall variation in spot and aurorae size and the relative sizes of spots versus aurorae respectively. Points denote PC scores for measured butterflies with colored points highlighting the specific populations analyzed in the current study. Panels (C), (D) and (E) show the proportion of the estimated additive genetic variation (Va) for each trait attributable to the autosomes (white region) versus the Z chromosome (orange region) for each population. Taxonomic designations for the three populations are: GNP = *L. idas*, SIN = *L. melissa*, and YG = *L. anna*.

We detected moderate to substantial contributions of additive genetic variation to wing pattern traits in each of the three *Lycaeides* populations analyzed (mean point estimates of the proportion of variation explained [PVE] were: GNP = 0.56, SIN = 0.72, and YG = 0.62; see Figure S27 for details, including uncertainty in estimates). In general, both the autosomes and the Z chromosome contributed to the PVE for wing pattern traits, with particularly large contributions from the Z for a subset of traits in specific populations (e.g., a2, a4, a5, and m in GNP, and a6 in YG) (Figure 6). Although the autosomes generally explained the majority of the explainable variance (mean = 90%), the Z chromosome’s contribution (10%) was disproportionately large relative to its size (the Z chromosome comprises 5.5% of the genome by size). Consequently, mixtures of ancestry for autosomes versus the Z chromosome is expected to influence wing pattern variation (and possibly other ecologically important traits, as we elaborate in the Discussion).

## Discussion

To what extent can we describe the evolutionary history of a taxonomic group with a strictly bifurcating tree consisting of well-delineated species? The answer to this question is important for framing our understanding of speciation, species, and the structure of the living world. Our results suggest that, at least for *Lycaeides* butterflies, the answer varies across the genome. We have shown that the genomes of these butterflies comprise a mosaic of genetic regions and chromosomes with distinct ancestries, such that no single tree is a sufficient summary of their evolutionary history. Moreover, we found that variation in evolutionary histories was of similar magnitude both within individual chromosomes and among different chromosomes. That said, the Z sex chromosome was exceptional, exhibiting a distinct and particularly tree-like history across the entire species complex. A similar pattern, an overall more tree-like history of a clade for the Z chromosome, was recently documented in *Heliconius* butterflies (Thawornwattana et al., 2023). We discuss our results and their broader implications in more detail below.

### Evolutionary history of *Lycaeides*

Bayesian phylogenetic inference indicates that *Lycaeides* first colonized Alaska and then spread southward roughly two million years ago (also see, e.g., Nice et al., 2005; Vila et al., 2011b). The nominal species *L. idas* appears to be paraphyletic and is likely the progenitor of both *L. anna* and *L. melissa* (and probably *L. samuelis*, though this remains to be tested), all of which are endemic to North America, specifically the USA and southern Canada. Our results strongly suggest that admixture has played a central role in the evolutionary history of the Sierra Nevada and Warner Mountain populations (consistent with, e.g., Gompert et al., 2006; Nice et al., 2013; Gompert et al., 2014), with substantial contributions from *L. melissa* and *L. anna*, as well as other ancestral populations more closely related to *L. anna* than to *L. melissa* (perhaps representing additional ancestral lineages of *L. idas*), as evidenced by these admixed populations sometimes forming distinct clades (e.g., for the Z chromosome). We found less, but still compelling, evidence of admixture in the Rocky Mountain populations. In particular, these populations harbor primarily *L. melissa* ancestry on the autosomes but *L. idas* ancestry on the Z chromosome. Past work suggested a larger genome-wide contribution of *L. idas* ancestry based on overall admixture proportions, but because those inferences relied on, or were heavily influenced by, ancestry-informative markers, they were likely disproportionately shaped by the Z chromosome (Gompert et al., 2010, 2012; Chaturvedi et al., 2020). Our results are also consistent with a North–South cline in the amount of *L. idas* ancestry in these populations, which likely reflects a combination of primary clinal divergence and post-Pleistocene secondary contact, with recent and ongoing hybridization at the geographic edges of this region facilitated by the spread of feral alfalfa (*Medicago sativa*), a host used by *L. melissa* (Gompert et al., 2010, 2012; Chaturvedi et al., 2020; Forister et al., 2020; Gompert et al., 2022). We have interpreted these patterns in the context of admixture (including primary divergence with gene flow), which we think is sensible given the large, chromosomal scale of the ancestry variation, the clinal structure, and direct evidence of ongoing hybridization (e.g., Gompert et al., 2010; Chaturvedi et al., 2020). However, it is also inevitable that at least some of the finer-scale variation among genomic regions reflects incomplete lineage sorting rather than admixture.

### Phenotypic consequences of admixture in *Lycaeides*

Gene flow among populations and species (i.e., admixture or introgressive hybridization) can generate novel phenotypes or adaptive trait combinations (e.g., Lewontin & Birch, 1966; Le Corre et al., 2020; Todesco et al., 2020; Meier et al., 2023; Rosser et al., 2024). By showing that both autosomes and the Z sex chromosome make major contributions to wing pattern traits–phenotypes that differ among *Lycaeides* species and contribute to mate choice (Fordyce et al., 2002; Gompert et al., 2013b; Lucas et al., 2018)–our results suggest that mosaic combinations of autosomal and Z-chromosome ancestry necessarily influence wing pattern variation in admixed populations (as would be expected for nearly any polygenic trait). This is also consistent with results from experimental crosses, which show that F1 hybrids exhibit mixtures of intermediate and transgressive wing patterns and male genitalic morphologies (another trait strongly distinguishing nominal species in this group) (Lucas et al., 2008, 2025). The role of novel combinations of mixed-ancestry autosomes and sex chromosomes for other ecologically important traits remains to be determined. Among such traits, diapause is of particular interest. *Lycaeides* diapause (overwinter) as neonate larvae inside eggs. Diapause is facultative in *L. melissa*, allowing multiple generations per year, whereas in *L. idas*, *L. anna* and all known admixed populations, it is obligate and only one generation occurs annually (Gompert et al., 2013b). In other Lepidoptera, obligate versus facultative diapause is controlled by known clock genes located on the Z chromosome (Jin et al., 2024). We do not yet know whether this is also true in *Lycaeides*, but the admixed populations analyzed here generally have greater *L. idas* or *L. anna* ancestry on the Z chromosome than *L. melissa* ancestry, consistent with this hypothesis. Regardless, our results to date suggest that the sorting of ancestry segments observed in admixed lineages has likely contributed to the novel, parental, intermediate, and transgressive phenotypes they possess.

### Implications for understanding species and speciation

From a whole-organism perspective, our results, combined with past work (Fordyce et al., 2002; Nice et al., 2002; Lucas et al., 2008; Gompert et al., 2013b; Lucas et al., 2018), suggest that *Lycaeides* are best viewed as a complex of recently diverged taxa in the early stages of the speciation process. Phenotypic and ecological differences exist among these taxa, along with a modest degree of pre- and postzygotic reproductive isolation (Fordyce et al., 2002; Gompert et al., 2013b; Lucas et al., 2025). Yet hybridization has been common in the past, and fertile hybrids are readily produced both in the lab and in natural hybrid zones (Gompert et al., 2006; Nice et al., 2013; Gompert et al., 2014; Chaturvedi et al., 2020; Lucas et al., 2025). However, speciation need not be viewed solely from a whole-organism perspective; instead, it can proceed at different rates across different regions of the genome, as evidenced by the semipermeable nature of species boundaries and emphasized by the genic view of speciation (Wu, 2001; Harrison & Larson, 2014; Westram et al., 2022). We think that such a genic view of speciation provides a useful context for interpreting the distinct histories and patterns of admixture for autosomes versus the Z sex chromosome in *Lycaeides*.

At least when gene flow is possible, progress toward speciation depends on an antagonism between divergent selection (which promotes speciation) and gene flow and recombination (which homogenize allele frequencies and break up locally adaptive gene complexes) (Felsenstein, 1981; Barton & Bengtsson, 1986; Flaxman et al., 2013). Thus, one expects genomic regions experiencing stronger selection or lower rates of recombination to behave more like species, that is, as distinct, independently evolving lineages. Several mechanisms could therefore cause sex chromosomes to effectively speciate before the rest of the genome, either because they have a disproportionate effect on hybrid fitness (stronger selection; i.e., the large X effect) or because of reduced recombination in the heterogametic sex (lower recombination) (Orr, 1997; Carling & Brumfield, 2008; Presgraves, 2008; Lenormand & Roze, 2025). Because recombination does not occur for any chromosomes (autosomes or sex chromosomes) in female butterflies (the heterogametic sex), and thus a greater proportion of Z chromosomes than autosomes have an opportunity for recombination (~2/3rds of Z chromosomes are in males, versus 1/2 of autosomes), we suspect that stronger selection, rather than reduced recombination, is responsible for the paucity of admixture in the history of the Z chromosome in *Lycaeides*.

More generally, the antagonism between selection and recombination suggests that introgression should be rarer in genomic regions where recombination rates are low or targets of selection are numerous (or selection is strong). Such patterns have been documented in several systems (see, e.g., Schumer et al., 2018) and are expected to result in more tree-like histories for these genomic regions. For example, recombination per base pair is often lower on longer chromosomes, leading to a commonly observed negative relationship between chromosome length and minor parent ancestry or introgression (e.g., Brandvain et al., 2014; Schumer et al., 2018; Martin et al., 2019; Chaturvedi et al., 2020; Nouhaud et al., 2022). Some of our results were consistent with this expectation; for instance, we found less evidence of introgression from *L. anna* (YG) or *L. melissa* (BHP) into the Sierra Nevada CP population on longer chromosomes. However, our TreeMix analyses suggested more tree-like histories (i.e., lower rates of admixture) for smaller chromosomes, which is the opposite of the expected pattern. Of course, other factors could contribute to variation in the outcomes of admixture, such as the effective population sizes (and thus genetic loads) of the hybridizing taxa and ecological selection favoring certain ancestry combinations (e.g., Gompert et al., 2012; Matute et al., 2020; Nouhaud et al., 2022; Springer et al., 2025).

At smaller scales, chromosomal rearrangements (especially inversions) can increase the strength of selection relative to recombination (particularly if they lock together co-adapted sets of alleles in supergenes), and thus may in some cases represent the leading edge of the speciation process. Previous work suggests that genomic regions harboring structural variation (in this case, primarily large deletions) are resistant to introgression in a *Lycaeides* hybrid zone, but we do not yet know the contribution of these chromosomal mutations to broader patterns of genomic variation in admixture and ancestry across this species complex (Zhang et al., 2023). Regardless of their specific relevance for *Lycaeides*, it is now clear that chromosomal rearrangements often underlie ecologically important differences between species as well as polymorphisms within species (e.g., Lowry & Willis, 2010; Koch et al., 2021; Akopyan et al., 2022; Nosil et al., 2023; Gompert et al., 2025). As such, these rearranged portions of the genome could function as evolutionarily distinct entities, behaving as species, before divergence occurs across much of the genome (in the case of polymorphic rearrangements, these evolutionary entities can occur within traditionally defined organismal populations). Such thinking advocates for a hierarchical view of biodiversity, encompassing various intragenomic evolutionary entities with different boundaries, histories, and degrees of evolutionary independence.

## Supplementary Material

Supplement 1

## Figures and Tables

**Table 1:** Population IDs, locations, geographic coordinates (in degrees) and sample sizes (*N*, in terms of number of diploid individuals) for the 22 *Lycaeides* populations (i.e., excluding *L. argyrognomon*, N = 10). Asterisks denote samples that were pooled and sequenced twice. The “Rockies” designation refers to Jackson Hole *Lycaeides* from past work (Gompert *et al*., 2012); “Sierra” includes the Sierra Nevada and CLH, which is from the nearby White Mountains.

Taxon	ID	Location	Longitude	Latitude	*N*

*L. anna*	LS	Leek Springs, CA	−120.24	38.63	48
	YG	Yuba Gap, CA	−120.60	39.32	48
	SHC	Shovel Creek, CA	−122.16	41.88	46
*L. idas*	GNP	Garnet Peak, MT	−111.22	45.43	56
	HNV	Hayden Valley, WY	−110.49	44.68	48
	TBY	Tomboy Rd., CO	−107.77	37.94	24
	SBW	Spruce Rd. & Barley Way, AK	−145.34	63.99	20
*L. melissa*	ABM	Albion Meadows, UT	−111.62	40.59	48
	BHP	Bishop, CA	−118.28	37.17	48
	HJ	Hallelujah Junction, CA	−120.07	39.78	48
	MTU	Montague, CA	−122.38	41.77	48
	SIN	Sinclair, WY	−107.93	41.85	48
	SUV	Surprise Valley, CA	−120.10	41.28	51
	VE	Verdi, NV	−120.00	39.51	48
Rockies	BTB	Blacktail Butte, WY	−110.68	43.64	48*
	BCR	Bull Creek, WY	−110.55	43.30	48*
Sierra	TIC	Tioga Crest, CA	−119.26	37.97	48
	CLH	County Line Hill, CA	−118.19	37.46	36
	CP	Carson Pass, CA	−120.02	38.71	48*
	MR	Mt. Rose, NV	−119.93	39.32	48
Warners	BKM	Buck Mt., CA	−120.29	41.69	33
	EP	Eagle Peak, CA	−120.22	41.26	48*

## Data Availability

The *Lycaeides* pooled whole genome sequence data have been deposited in the NCBI SRA (accession pending). Wing pattern data are available from Dryad (https://doi.org/10.5061/dryad.fc827). Code and associated documentation is available at https://github.com/zgompert/LycAdmixMosaic. Final versions of computer code will be deposited on Zendo when the manuscript is accepted.

## Teaser text:

How well do bifurcating trees comprising distinct species capture the evolutionary histories of groups of organisms? Answering this question is central to our understanding of species, speciation, and the organization of biological diversity. We show that, at least for *Lycaeides* butterflies, the answer differs for different parts of the genome. Our results suggest that these butterflies’ genomes comprise a mixture of genomic regions and chromosomes with distinct evolutionary histories. In particular, we found substantial evidence of historical hybridization and mixed ancestry on the autosomes, but distinct and more tree-like histories on the Z sex chromosome. Our genetic mapping results indicate that these different histories are relevant for polygenic trait variation, specifically wing patterns, which play a central role in mate preference. Thus, our results shed light on the multifaceted nature of species and the sources of variation that fuel evolution.
