# Supplementary material for: Admixture in a butterfly species complex creates a genomic mosaic of ancestry with distinct histories for different chromosomes
Source: bioRxiv. 2025 Nov 29:2025.11.28.691233. Preprint. [Version 1] doi: 10.1101/2025.11.28.691233 (PMC12704007; doi:10.1101/2025.11.28.691233)
Supplement: Supplement 1 [file NIHPP2025.11.28.691233v1-supplement-1.pdf]

bioRxiv preprint doi: https://doi.org/10.1101/2025.11.28.691233; this version posted November 29, 2025. The copyright holder for this preprint (which was not certified by peer review) is the author/funder, who has granted bioRxiv a license to display the preprint in perpetuity. It is made available under aCC-BY 4.0 International license.

Online Supplemental Materials for: Admixture in a butterfly species complex creates a genomic mosaic of ancestry with distinct histories for different chromosomes

Supplemental Methods

Library preparation, sequencing and read filtering

Sequencing libraries were constructed by BGI. In brief, Genomic DNA was fragmented with a Covaris ultrasonicator and fragment size distributions were verified on an Agilent 2100 Bioanalyzer. Fragmented DNA underwent end-repair and 3' dA-tailing followed by bead-based cleanup. Indexed adapters were ligated to A-tailed fragments and ligation products were purified with magnetic beads. Adapter-ligated DNA was then amplified with KAPA HiFi HotStart DNA Polymerase. Amplified libraries were then converted to single-stranded circular DNA using an MGI circularization workflow. DNA nanoballs were generated from the single-stranded circles by rolling-circle amplification. Sequencing was performed using the DNBseq platform with paired-end 100 bp reads and a target coverage of 100× for each library. After sequencing, the raw reads were filtered. Data filtering was accomplished with `SOAPnuke` and included removing adaptor sequences, contamination and low-quality reads from raw reads. Specifically, adapters were removed if reads matched 25% or more of the adapter sequence (with a 2 bp maximum mismatch) and reads were discarded if 40% or more of the bases had quality scores less than 10 or if Ns comprised more than 0.1% of the read (i.e., $\geq$1 N).

Alignment, variant calling, filtering and allele frequency estimation

We aligned the sequence reads to the *L. melissa* genome using `bwa-mem2` (version 2.0pre2) with default parameters (Li & Durbin, 2009; Vasimuddin *et al.*, 2019). PCR duplicates were

bioRxiv preprint doi: https://doi.org/10.1101/2025.11.28.691233; this version posted November 29, 2025. The copyright holder for this preprint (which was not certified by peer review) is the author/funder, who has granted bioRxiv a license to display the preprint in perpetuity. It is made available under aCC-BY 4.0 International license.

then marked and removed with `samtools` (version 1.16) using the `collate`, `fixmate` and `markdup` commands (Li *et al.*, 2009; Ebbert *et al.*, 2016). We called genetic variants using `bcftools` consensus caller (option `-c`) (version 1.16) (Li, 2011). For this, we skipped alignments with mapping quality less than 20 and bases with quality scores less than 30, ignored insertion-deletion polymorphisms, and only called SNPs if the probability all populations were fixed for the reference allele given the data was less than 0.01. We then filtered the initial SNP set to retain only SNPs that were bi-allelic, had qualities >30, total read coverage >1350, and base-quality bias, mapping-quality bias, and read-position bias Z-scores $< \pm 3$. We used `GATK` (version 4.1.4.1) (McKenna *et al.*, 2010) for filtering.

We extracted the counts of high-quality reads supporting the reference and alternate allele for each SNP and population. We used these counts to obtain maximum likelihood estimates of allele frequencies, which are equivalent to the sample allele frequencies computed from the read counts. This was done in `R`. We used these allele frequency estimates for principal component analysis (PCA) and for generating input sequences for phylogenetic analyses, as described in the next section. For PCA, we used non-reference allele frequencies of 0.001 for populations with no reads (fully missing data) for a given SNP.

Phylogenetic model and priors

We began by characterizing the overall relationships among the *Lycaeides* populations. We took two complementary approaches. First, we used `Beast` (version 2.7.7) to estimate a time-calibrated phylogeny (a chronogram) from a subset of genome-wide SNPs. Specifically, we used a concatenated set of 5408 SNPs interspersed across the genome (as required for computational feasibility); variable sites within populations were coded as the more common allele when its frequency was ≥ 0.95 and as ambiguous (N) when this was not true. Invariant sites were also accounted for in the model. We used existing estimates for the divergence of *L. argyrognomon* and North American *Lycaeides* and for the colonization of North America by *Lycaeides* to construct informative priors for the molecular clock in our analysis (Vila *et al.*,

bioRxiv preprint doi: https://doi.org/10.1101/2025.11.28.691233; this version posted November 29, 2025. The copyright holder for this preprint (which was not certified by peer review) is the author/funder, who has granted bioRxiv a license to display the preprint in perpetuity. It is made available under aCC-BY 4.0 International license.

2011b; Kawahara *et al.*, 2023). See the OSM for a detailed description of the phylogenetic model and priors uses. Second, we summarized patterns of genetic variation and genetic similarity using a principal components analysis (PCA) of genome-wide allele frequencies. We did this in R. We separately analyzed the 14,907,399 autosomal SNPs and the 1,007,427 Z chromosome SNPs. In both cases, we included only the *Lycaeides* populations (i.e., we excluded the outgroup, *L. argyrognomon*) and analyzed the centered, but not standardized, allele frequency matrix.

Bayesian regression methods

We then tested for a relationship between chromosome size (in base pairs) and the proportion of covariance explained by trees and graphs with a given number of migration edges. Assuming recombination per base pair is lower on longer chromosomes (i.e., that recombination per chromosome is approximately constant) and that selection against deleterious foreign alleles removes larger DNA blocks on longer chromosomes (e.g., Brandvain *et al.*, 2014; Schumer *et al.*, 2018), we might expect allele-frequency covariances to be better explained by a bifurcating tree (or a graph with only a few admixture events) for short chromosomes than for long chromosomes. In contrast, we might expect no such relationship with chromosome size for admixture graphs with many migration events. We fit Bayesian regression models to test for these patterns, as described in the OSM. Finally, we examined the nature and weights–proxies for admixture proportions–of the migration edges across chromosomes and graphs to identify the most noteworthy instances of putative admixture.

DNA sequence alignment and variant calling, and genetic mapping

We last asked whether differential evolutionary histories and ancestry for autosomes versus the Z chromosome (as documented in the Results) would be expected to have meaningful phenotypic consequences for ecologically important traits in admixed populations. We used

bioRxiv preprint doi: https://doi.org/10.1101/2025.11.28.691233; this version posted November 29, 2025. The copyright holder for this preprint (which was not certified by peer review) is the author/funder, who has granted bioRxiv a license to display the preprint in perpetuity. It is made available under aCC-BY 4.0 International license.

wing pattern as a test case for this, and specifically asked whether both autosomes and the Z sex chromosome contributed substantially to wing pattern variation. *Lycaeides* wing patterns comprise a series of black spots and aurorae (border ocelli with black and orange pigments, as well as structural colors) (Nabokov, 1943; Fordyce *et al.*, 2002; Lucas *et al.*, 2018). We re-analyzed wing pattern measurements (the sizes of 17 wing pattern elements) and corresponding genetic data from three *Lycaeides* populations: YG (*L. anna*, $n = 100$), GNP (*L. idas*, $n = 98$), and SIN (*L. melissa*, $n = 97$) (Lucas *et al.*, 2018). We used a polygenic genome-wide association mapping approach to estimate the proportion of the additive genetic variance explained for each trait by each chromosome in each of the three populations (Zhou *et al.*, 2013). This was done by fitting Bayesian sparse linear mixed models in `gemma` (version 0.95a) (Zhou & Stephens, 2012). We ran 20 chains of 1,000,000 iterations (500,000 iteration burnins) for each trait and population, and estimated additive genetic variances from the model-averaged SNP effect estimates and population allele frequencies. Details of these data, DNA sequence alignment and variant calling, and genetic mapping are provided in the OSM.

Supplemental Figures

bioRxiv preprint doi: https://doi.org/10.1101/2025.11.28.691233; this version posted November 29, 2025. The copyright holder for this preprint (which was not certified by peer review) is the author/funder, who has granted bioRxiv a license to display the preprint in perpetuity. It is made available under aCC-BY 4.0 International license.

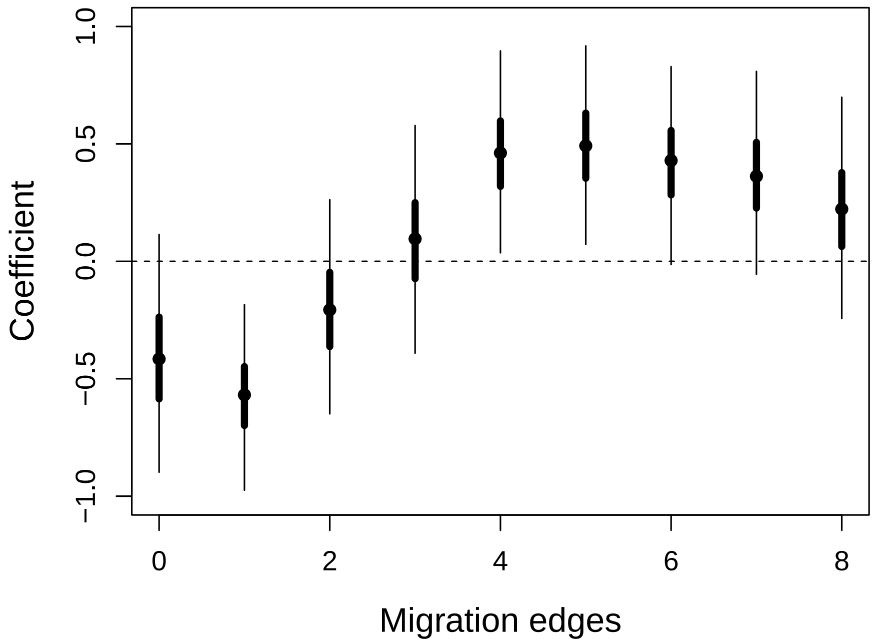

Figure S1: Bayesian estimates of regression coefficients for the association of chromosome size the the proportion of variation explained by a tree or graph with a given number of migration edges. Posteriors are summarized in terms of the median (points), 50% equal-tail probability intervals [ETPIs] (thick lines), and 95% ETPIs (thin lines). The horizontal dashed line denotes a regression coefficient of zero, that is, no effect of chromosome size on the covariance explained.

bioRxiv preprint doi: https://doi.org/10.1101/2025.11.28.691233; this version posted November 29, 2025. The copyright holder for this preprint (which was not certified by peer review) is the author/funder, who has granted bioRxiv a license to display the preprint in perpetuity. It is made available under aCC-BY 4.0 International license.

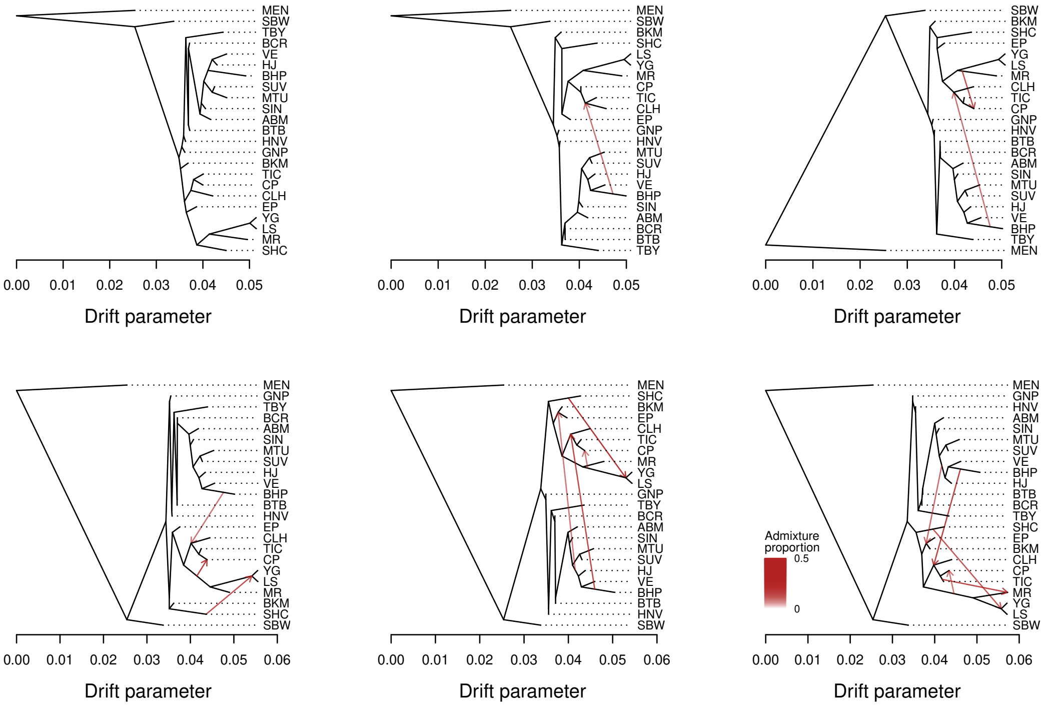

Figure S2: Population graphs for chromosome 1, with units in terms of a drift parameter proportional to evolutionary change and with the *L. argyrognomon* population (MEN) included as an outgroup. Graphs are shown for m = 0 (bifurcating tree) to five migration edges (red arrows). The admixture proportions associated with each are indicated by the intensity of each red arrow.

bioRxiv preprint doi: https://doi.org/10.1101/2025.11.28.691233; this version posted November 29, 2025. The copyright holder for this preprint (which was not certified by peer review) is the author/funder, who has granted bioRxiv a license to display the preprint in perpetuity. It is made available under aCC-BY 4.0 International license.

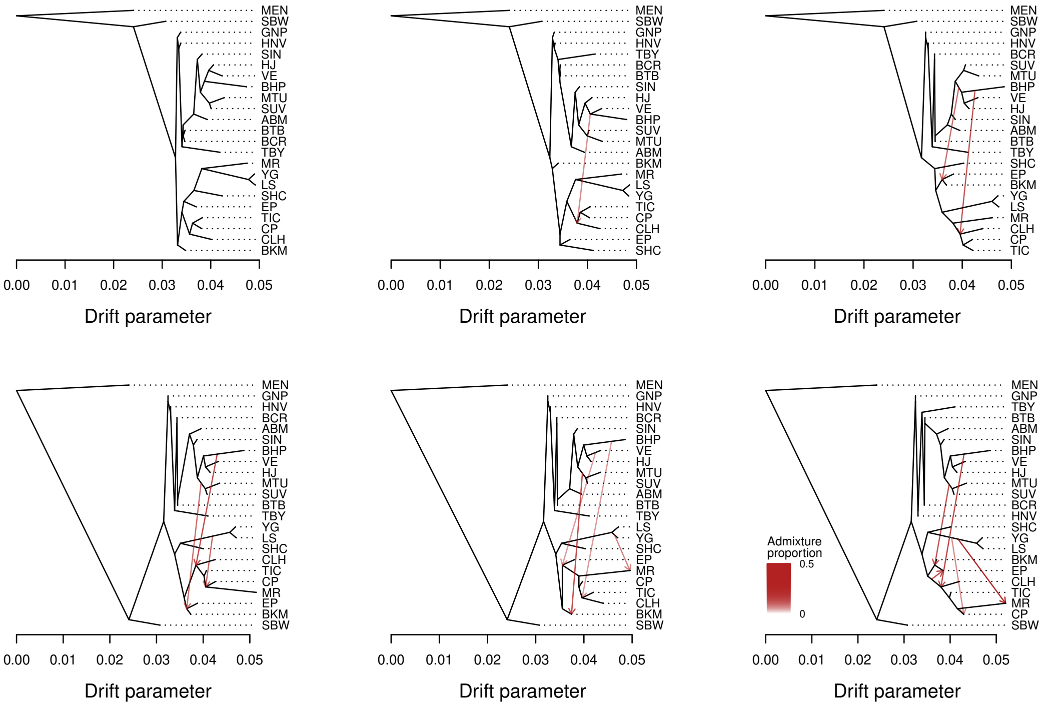

Figure S3: Population graphs for chromosome 2, with units in terms of a drift parameter proportional to evolutionary change and with the *L. argyrognomon* population (MEN) included as an outgroup. Graphs are shown for m = 0 (bifurcating tree) to five migration edges (red arrows). The admixture proportions associated with each are indicated by the intensity of each red arrow.

bioRxiv preprint doi: https://doi.org/10.1101/2025.11.28.691233; this version posted November 29, 2025. The copyright holder for this preprint (which was not certified by peer review) is the author/funder, who has granted bioRxiv a license to display the preprint in perpetuity. It is made available under aCC-BY 4.0 International license.

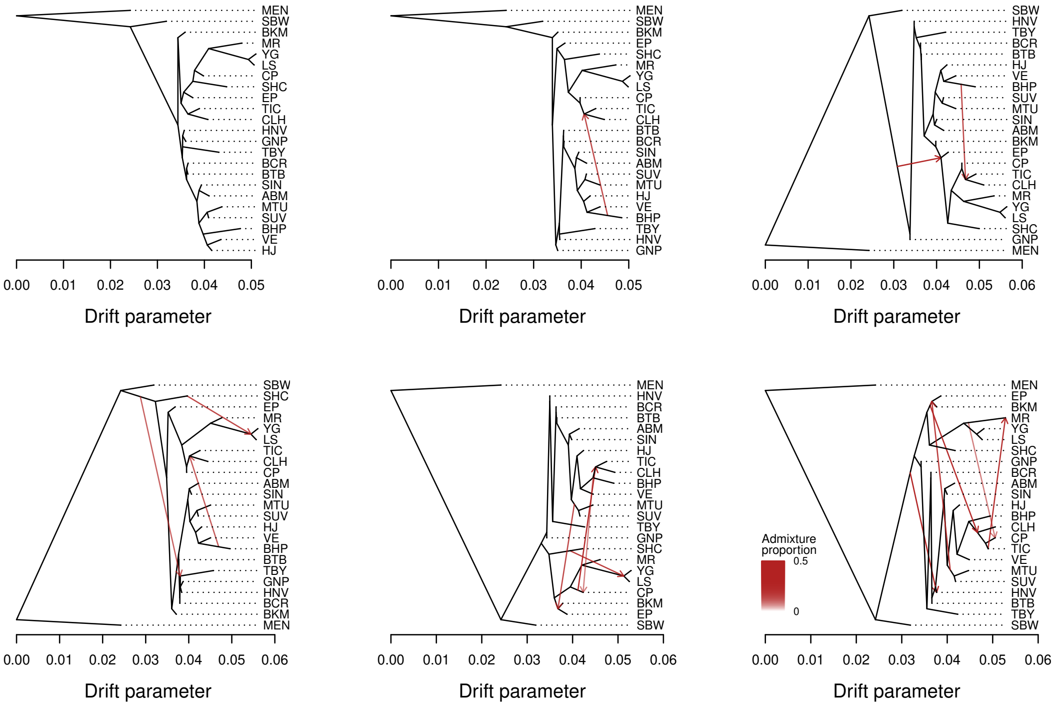

Figure S4: Population graphs for chromosome 3, with units in terms of a drift parameter proportional to evolutionary change and with the *L. argyrognomon* population (MEN) included as an outgroup. Graphs are shown for m = 0 (bifurcating tree) to five migration edges (red arrows). The admixture proportions associated with each are indicated by the intensity of each red arrow.

bioRxiv preprint doi: https://doi.org/10.1101/2025.11.28.691233; this version posted November 29, 2025. The copyright holder for this preprint (which was not certified by peer review) is the author/funder, who has granted bioRxiv a license to display the preprint in perpetuity. It is made available under aCC-BY 4.0 International license.

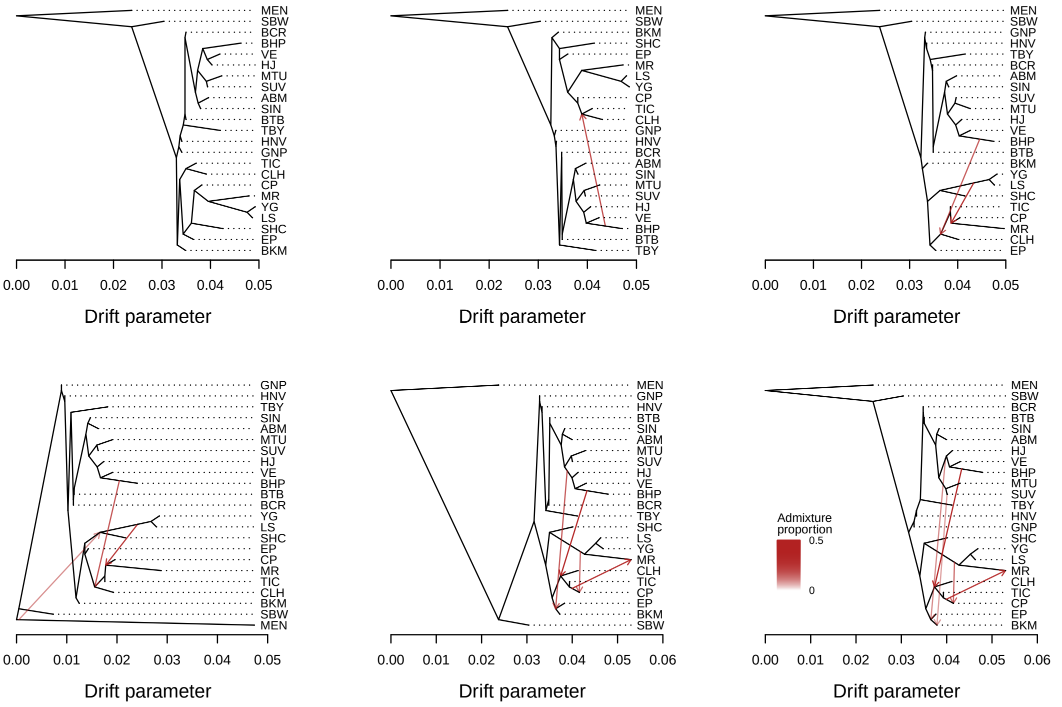

Figure S5: Population graphs for chromosome 4, with units in terms of a drift parameter proportional to evolutionary change and with the *L. argyrognomon* population (MEN) included as an outgroup. Graphs are shown for m = 0 (bifurcating tree) to five migration edges (red arrows). The admixture proportions associated with each are indicated by the intensity of each red arrow.

bioRxiv preprint doi: https://doi.org/10.1101/2025.11.28.691233; this version posted November 29, 2025. The copyright holder for this preprint (which was not certified by peer review) is the author/funder, who has granted bioRxiv a license to display the preprint in perpetuity. It is made available under aCC-BY 4.0 International license.

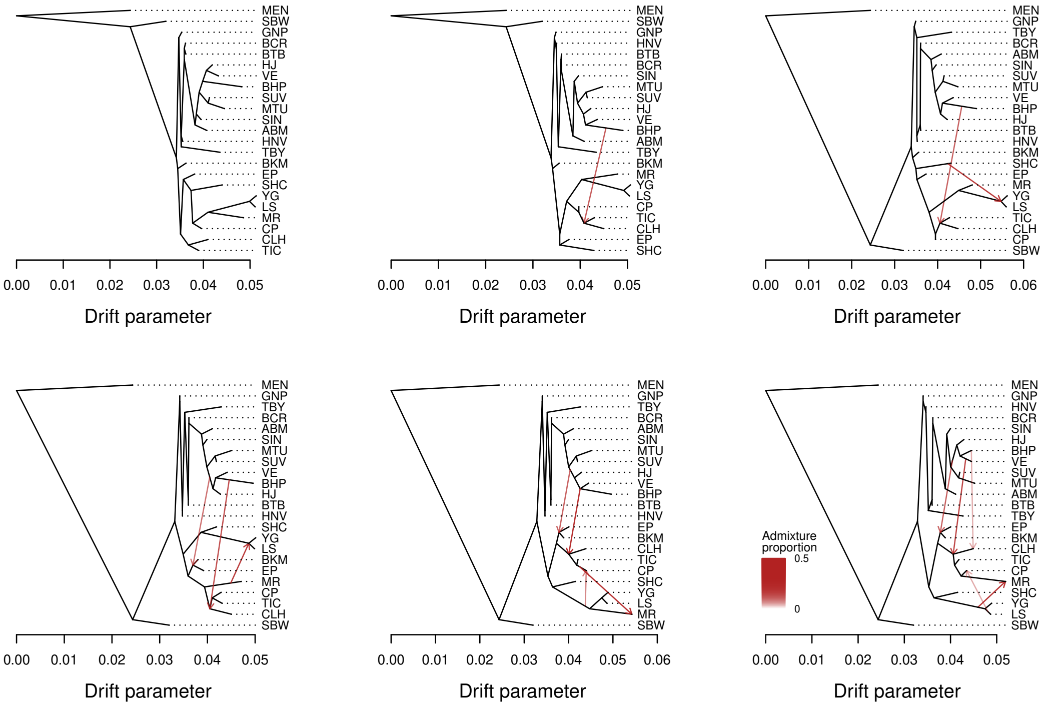

Figure S6: Population graphs for chromosome 5, with units in terms of a drift parameter proportional to evolutionary change and with the *L. argyrognomon* population (MEN) included as an outgroup. Graphs are shown for m = 0 (bifurcating tree) to five migration edges (red arrows). The admixture proportions associated with each are indicated by the intensity of each red arrow.

bioRxiv preprint doi: https://doi.org/10.1101/2025.11.28.691233; this version posted November 29, 2025. The copyright holder for this preprint (which was not certified by peer review) is the author/funder, who has granted bioRxiv a license to display the preprint in perpetuity. It is made available under aCC-BY 4.0 International license.

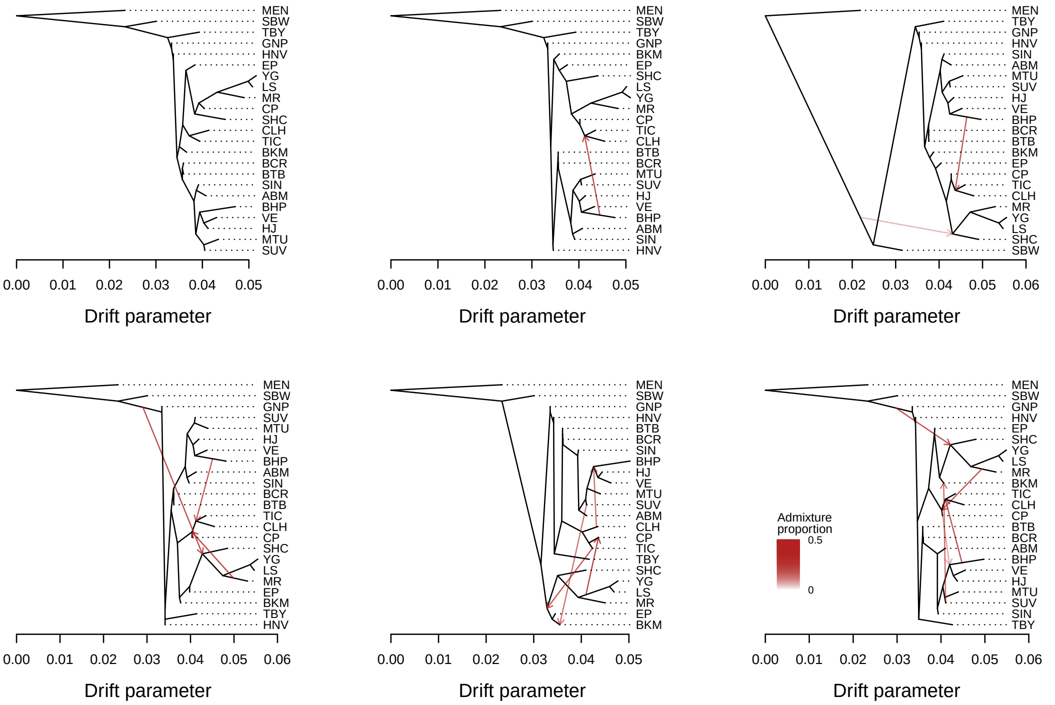

Figure S7: Population graphs for chromosome 6, with units in terms of a drift parameter proportional to evolutionary change and with the *L. argyrognomon* population (MEN) included as an outgroup. Graphs are shown for m = 0 (bifurcating tree) to five migration edges (red arrows). The admixture proportions associated with each are indicated by the intensity of each red arrow.

bioRxiv preprint doi: https://doi.org/10.1101/2025.11.28.691233; this version posted November 29, 2025. The copyright holder for this preprint (which was not certified by peer review) is the author/funder, who has granted bioRxiv a license to display the preprint in perpetuity. It is made available under aCC-BY 4.0 International license.

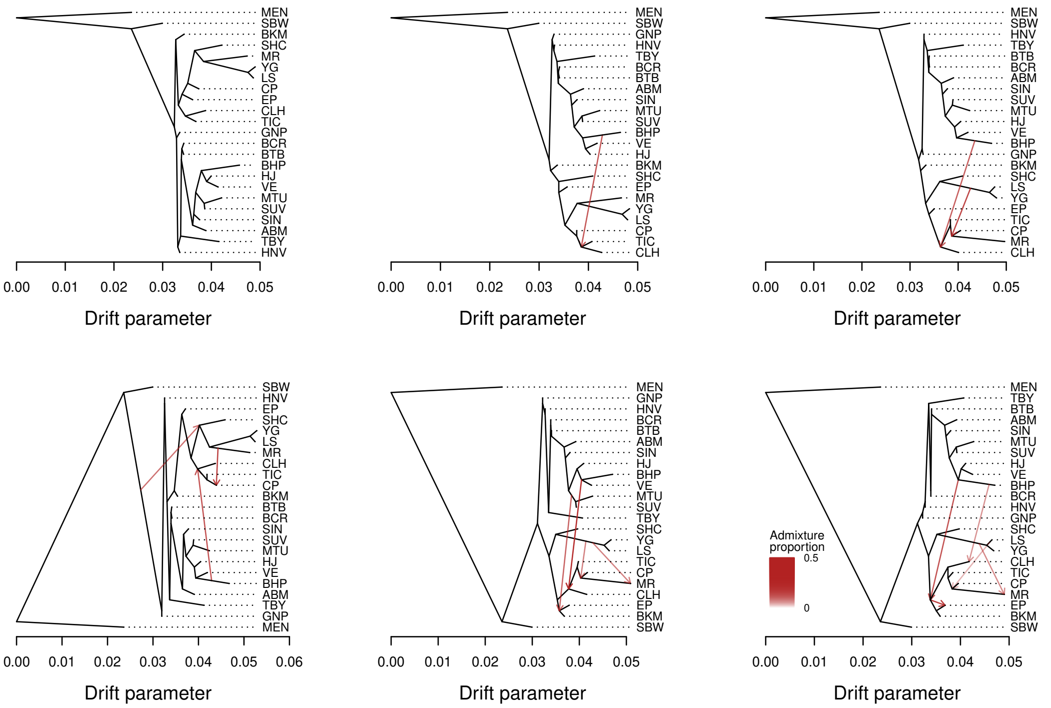

Figure S8: Population graphs for chromosome 7, with units in terms of a drift parameter proportional to evolutionary change and with the *L. argyrognomon* population (MEN) included as an outgroup. Graphs are shown for m = 0 (bifurcating tree) to five migration edges (red arrows). The admixture proportions associated with each are indicated by the intensity of each red arrow.

bioRxiv preprint doi: https://doi.org/10.1101/2025.11.28.691233; this version posted November 29, 2025. The copyright holder for this preprint (which was not certified by peer review) is the author/funder, who has granted bioRxiv a license to display the preprint in perpetuity. It is made available under aCC-BY 4.0 International license.

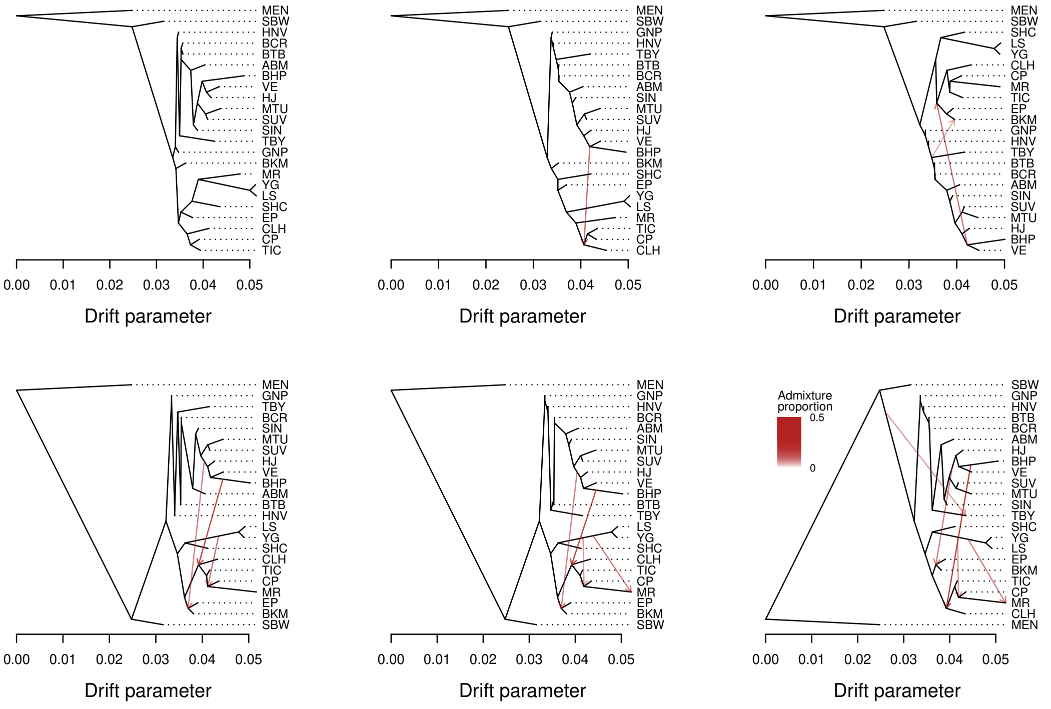

Figure S9: Population graphs for chromosome 8, with units in terms of a drift parameter proportional to evolutionary change and with the *L. argyrognomon* population (MEN) included as an outgroup. Graphs are shown for m = 0 (bifurcating tree) to five migration edges (red arrows). The admixture proportions associated with each are indicated by the intensity of each red arrow.

bioRxiv preprint doi: https://doi.org/10.1101/2025.11.28.691233; this version posted November 29, 2025. The copyright holder for this preprint (which was not certified by peer review) is the author/funder, who has granted bioRxiv a license to display the preprint in perpetuity. It is made available under aCC-BY 4.0 International license.

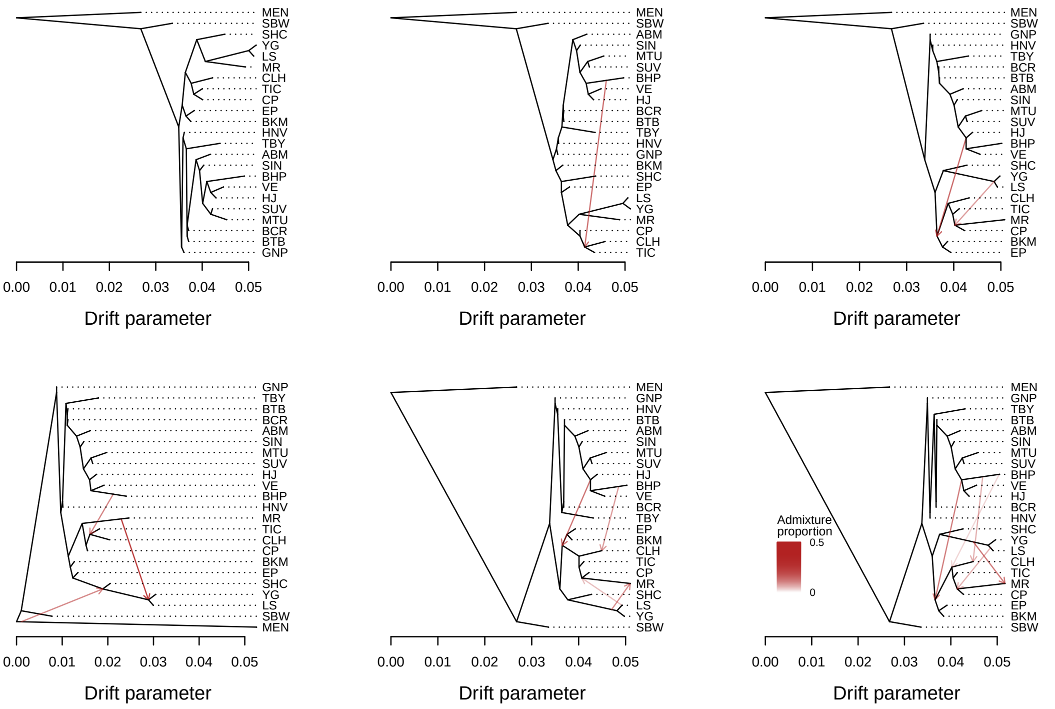

Figure S10: Population graphs for chromosome 9, with units in terms of a drift parameter proportional to evolutionary change and with the *L. argyrognomon* population (MEN) included as an outgroup. Graphs are shown for m = 0 (bifurcating tree) to five migration edges (red arrows). The admixture proportions associated with each are indicated by the intensity of each red arrow.

bioRxiv preprint doi: https://doi.org/10.1101/2025.11.28.691233; this version posted November 29, 2025. The copyright holder for this preprint (which was not certified by peer review) is the author/funder, who has granted bioRxiv a license to display the preprint in perpetuity. It is made available under aCC-BY 4.0 International license.

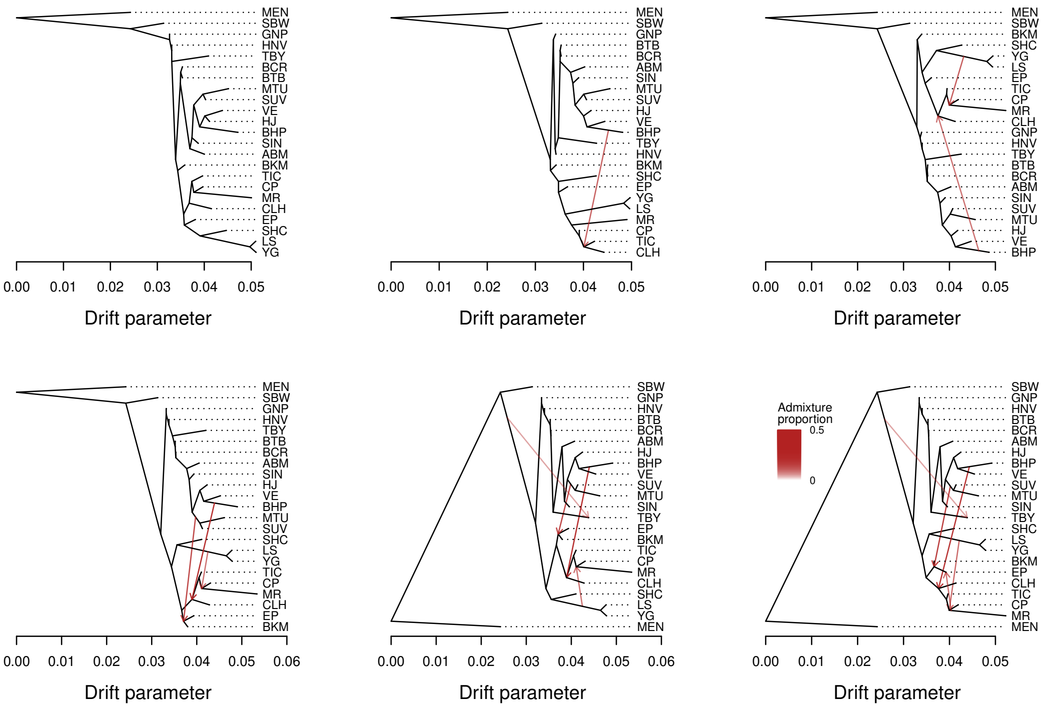

Figure S11: Population graphs for chromosome 10, with units in terms of a drift parameter proportional to evolutionary change and with the *L. argyrognomon* population (MEN) included as an outgroup. Graphs are shown for m = 0 (bifurcating tree) to five migration edges (red arrows). The admixture proportions associated with each are indicated by the intensity of each red arrow.

bioRxiv preprint doi: https://doi.org/10.1101/2025.11.28.691233; this version posted November 29, 2025. The copyright holder for this preprint (which was not certified by peer review) is the author/funder, who has granted bioRxiv a license to display the preprint in perpetuity. It is made available under aCC-BY 4.0 International license.

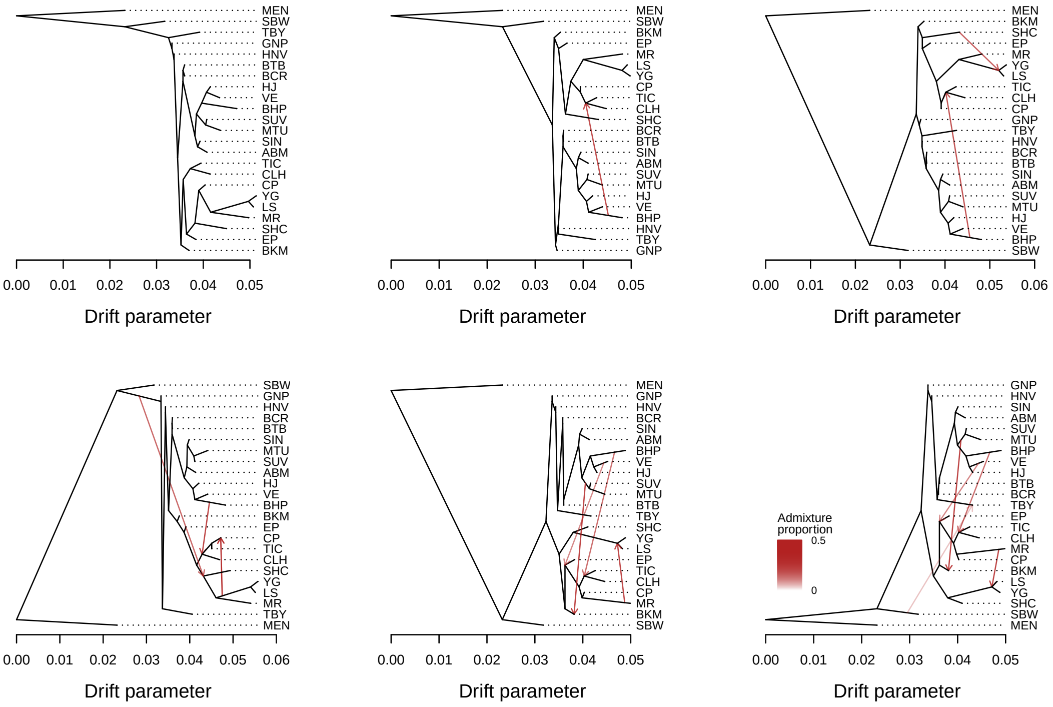

Figure S12: Population graphs for chromosome 11, with units in terms of a drift parameter proportional to evolutionary change and with the *L. argyrognomon* population (MEN) included as an outgroup. Graphs are shown for m = 0 (bifurcating tree) to five migration edges (red arrows). The admixture proportions associated with each are indicated by the intensity of each red arrow.

bioRxiv preprint doi: https://doi.org/10.1101/2025.11.28.691233; this version posted November 29, 2025. The copyright holder for this preprint (which was not certified by peer review) is the author/funder, who has granted bioRxiv a license to display the preprint in perpetuity. It is made available under aCC-BY 4.0 International license.

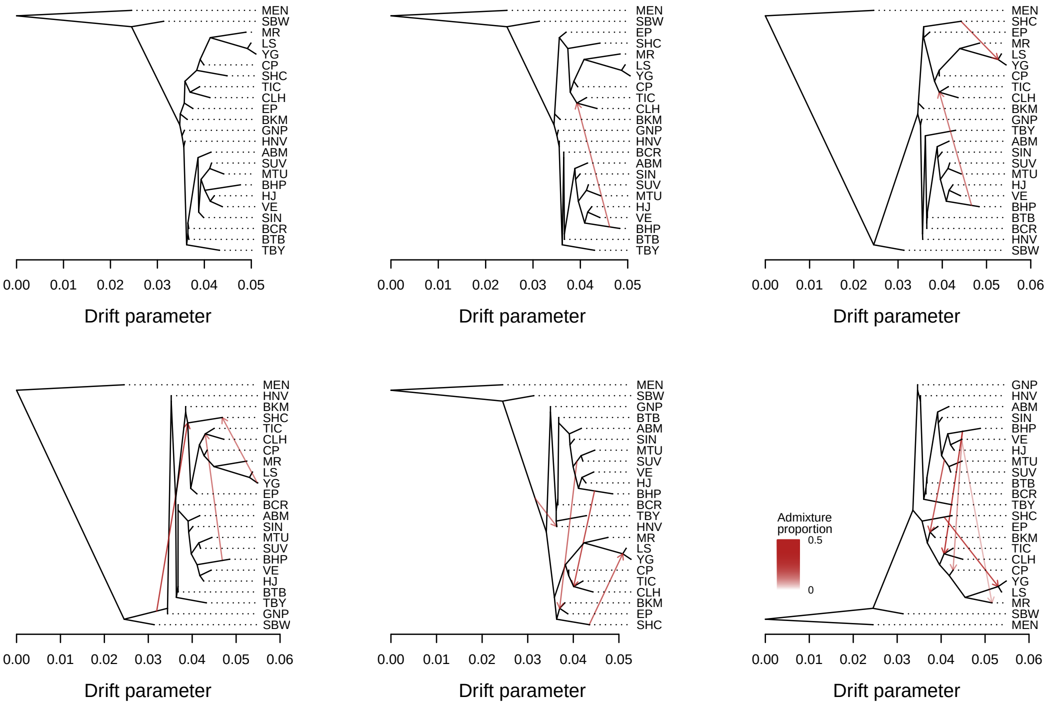

Figure S13: Population graphs for chromosome 12, with units in terms of a drift parameter proportional to evolutionary change and with the *L. argyrognomon* population (MEN) included as an outgroup. Graphs are shown for m = 0 (bifurcating tree) to five migration edges (red arrows). The admixture proportions associated with each are indicated by the intensity of each red arrow.

bioRxiv preprint doi: https://doi.org/10.1101/2025.11.28.691233; this version posted November 29, 2025. The copyright holder for this preprint (which was not certified by peer review) is the author/funder, who has granted bioRxiv a license to display the preprint in perpetuity. It is made available under aCC-BY 4.0 International license.

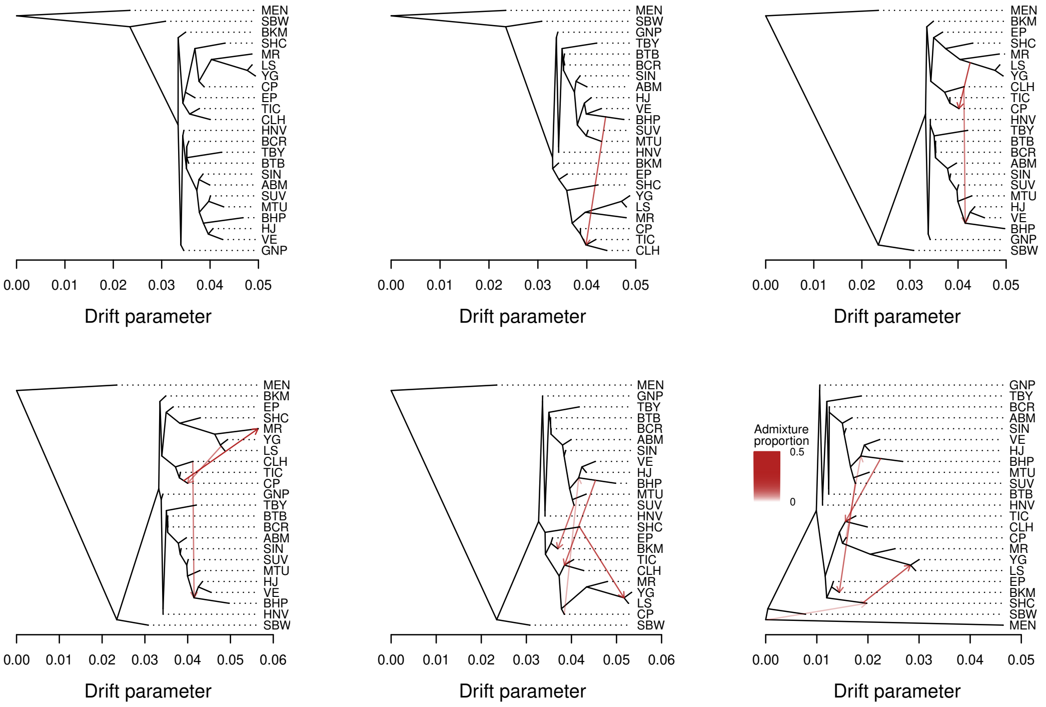

Figure S14: Population graphs for chromosome 13, with units in terms of a drift parameter proportional to evolutionary change and with the *L. argyrognomon* population (MEN) included as an outgroup. Graphs are shown for m = 0 (bifurcating tree) to five migration edges (red arrows). The admixture proportions associated with each are indicated by the intensity of each red arrow.

bioRxiv preprint doi: https://doi.org/10.1101/2025.11.28.691233; this version posted November 29, 2025. The copyright holder for this preprint (which was not certified by peer review) is the author/funder, who has granted bioRxiv a license to display the preprint in perpetuity. It is made available under aCC-BY 4.0 International license.

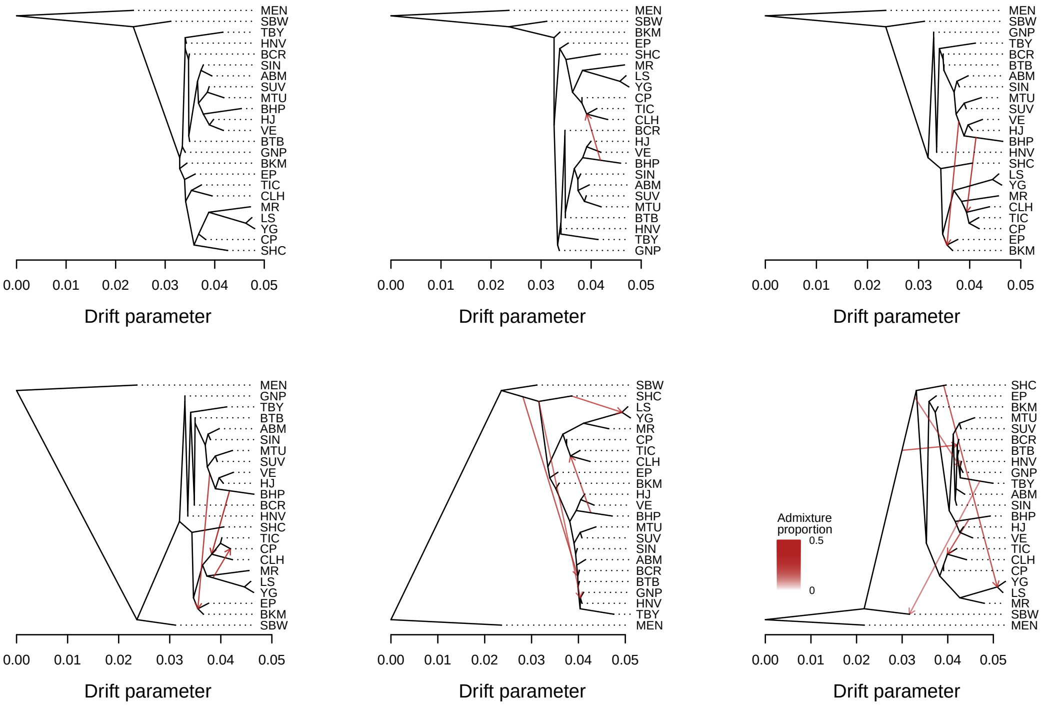

Figure S15: Population graphs for chromosome 14, with units in terms of a drift parameter proportional to evolutionary change and with the *L. argyrognomon* population (MEN) included as an outgroup. Graphs are shown for m = 0 (bifurcating tree) to five migration edges (red arrows). The admixture proportions associated with each are indicated by the intensity of each red arrow.

bioRxiv preprint doi: https://doi.org/10.1101/2025.11.28.691233; this version posted November 29, 2025. The copyright holder for this preprint (which was not certified by peer review) is the author/funder, who has granted bioRxiv a license to display the preprint in perpetuity. It is made available under aCC-BY 4.0 International license.

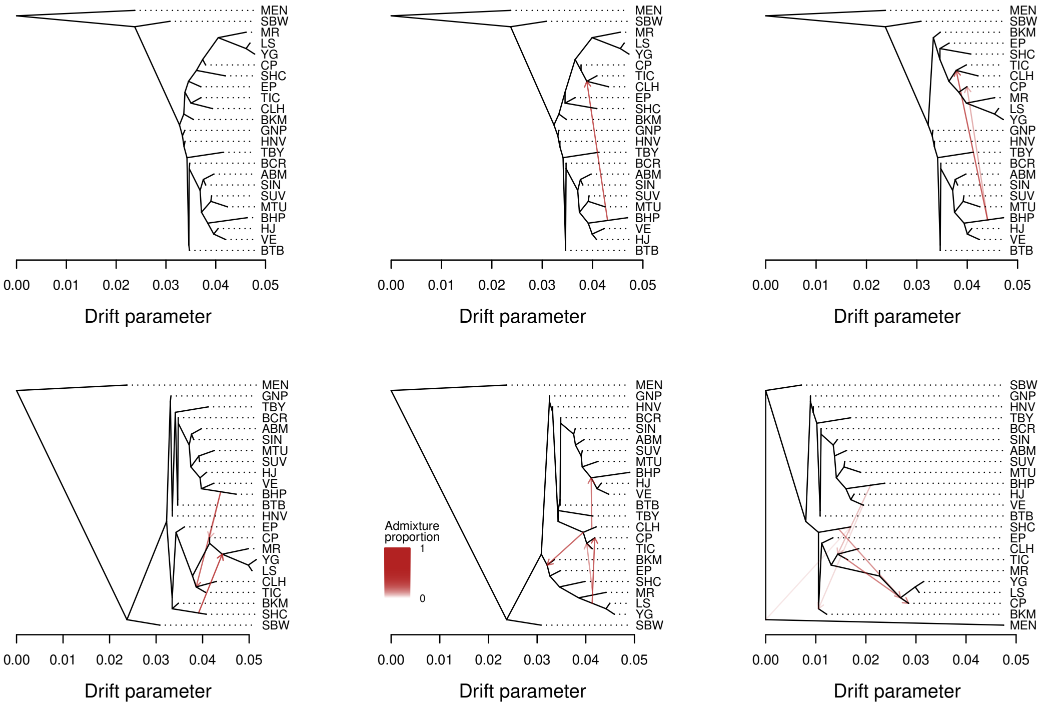

Figure S16: Population graphs for chromosome 15, with units in terms of a drift parameter proportional to evolutionary change and with the *L. argyrognomon* population (MEN) included as an outgroup. Graphs are shown for m = 0 (bifurcating tree) to five migration edges (red arrows). The admixture proportions associated with each are indicated by the intensity of each red arrow.

bioRxiv preprint doi: https://doi.org/10.1101/2025.11.28.691233; this version posted November 29, 2025. The copyright holder for this preprint (which was not certified by peer review) is the author/funder, who has granted bioRxiv a license to display the preprint in perpetuity. It is made available under aCC-BY 4.0 International license.

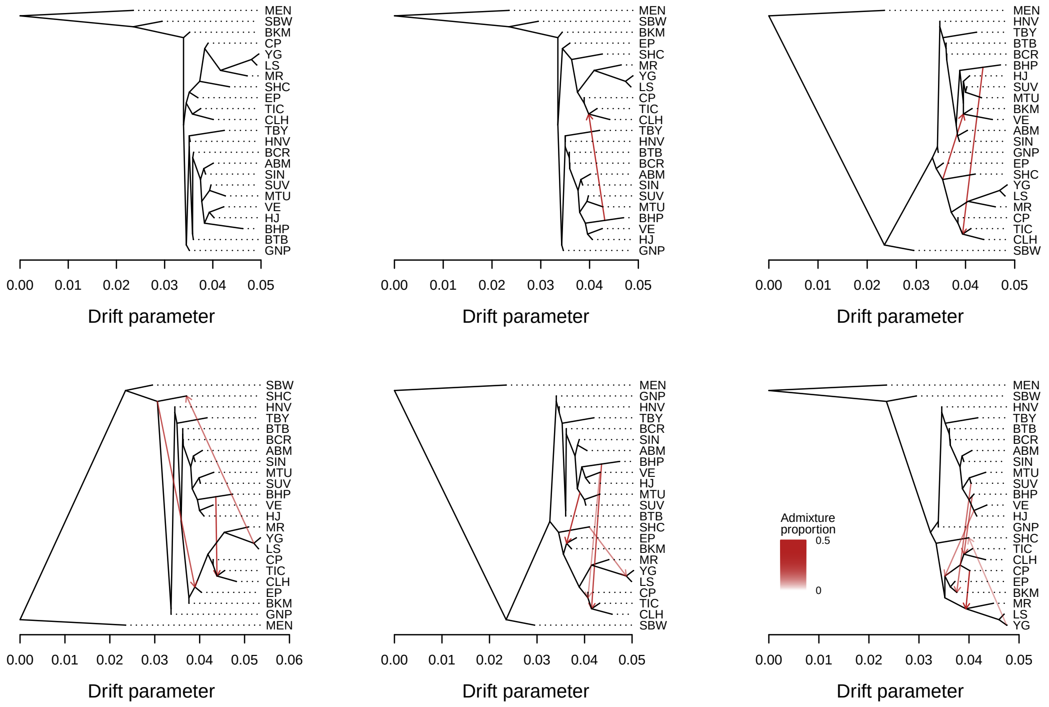

Figure S17: Population graphs for chromosome 16, with units in terms of a drift parameter proportional to evolutionary change and with the *L. argyrognomon* population (MEN) included as an outgroup. Graphs are shown for m = 0 (bifurcating tree) to five migration edges (red arrows). The admixture proportions associated with each are indicated by the intensity of each red arrow.

bioRxiv preprint doi: https://doi.org/10.1101/2025.11.28.691233; this version posted November 29, 2025. The copyright holder for this preprint (which was not certified by peer review) is the author/funder, who has granted bioRxiv a license to display the preprint in perpetuity. It is made available under aCC-BY 4.0 International license.

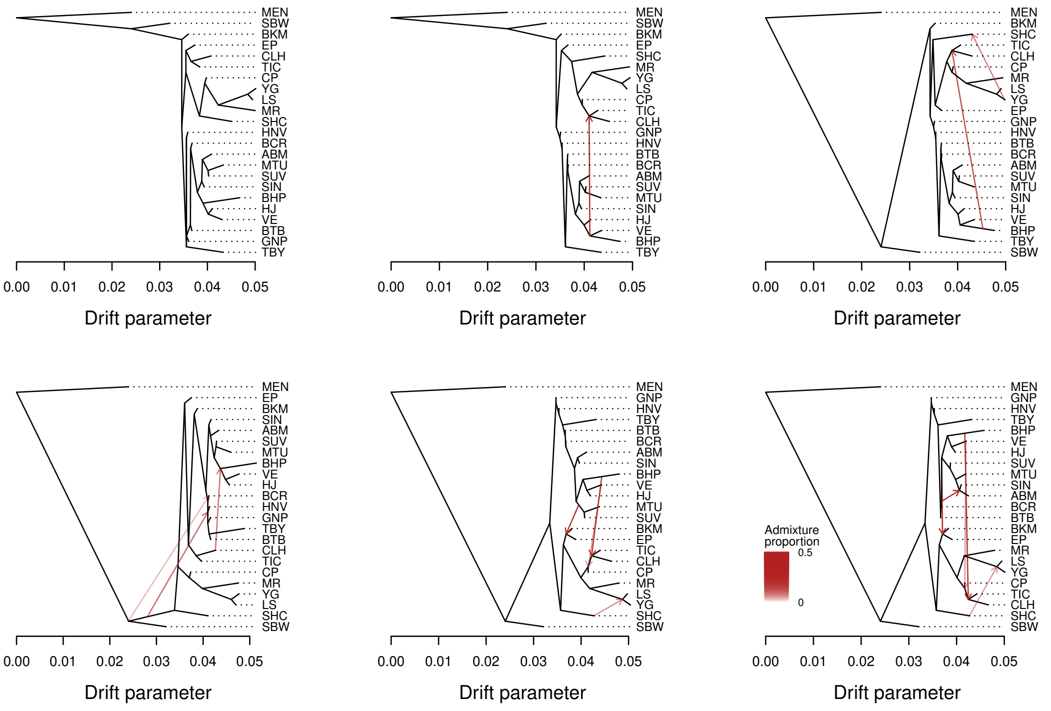

Figure S18: Population graphs for chromosome 17, with units in terms of a drift parameter proportional to evolutionary change and with the *L. argyrognomon* population (MEN) included as an outgroup. Graphs are shown for m = 0 (bifurcating tree) to five migration edges (red arrows). The admixture proportions associated with each are indicated by the intensity of each red arrow.

bioRxiv preprint doi: https://doi.org/10.1101/2025.11.28.691233; this version posted November 29, 2025. The copyright holder for this preprint (which was not certified by peer review) is the author/funder, who has granted bioRxiv a license to display the preprint in perpetuity. It is made available under aCC-BY 4.0 International license.

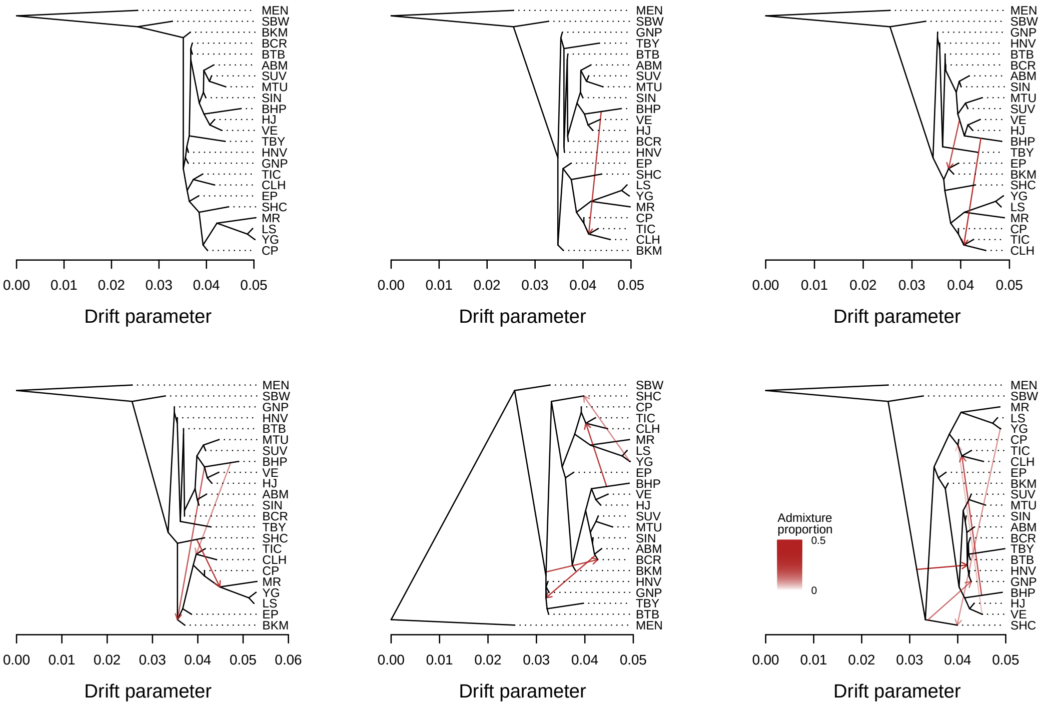

Figure S19: Population graphs for chromosome 18, with units in terms of a drift parameter proportional to evolutionary change and with the *L. argyrognomon* population (MEN) included as an outgroup. Graphs are shown for m = 0 (bifurcating tree) to five migration edges (red arrows). The admixture proportions associated with each are indicated by the intensity of each red arrow.

bioRxiv preprint doi: https://doi.org/10.1101/2025.11.28.691233; this version posted November 29, 2025. The copyright holder for this preprint (which was not certified by peer review) is the author/funder, who has granted bioRxiv a license to display the preprint in perpetuity. It is made available under aCC-BY 4.0 International license.

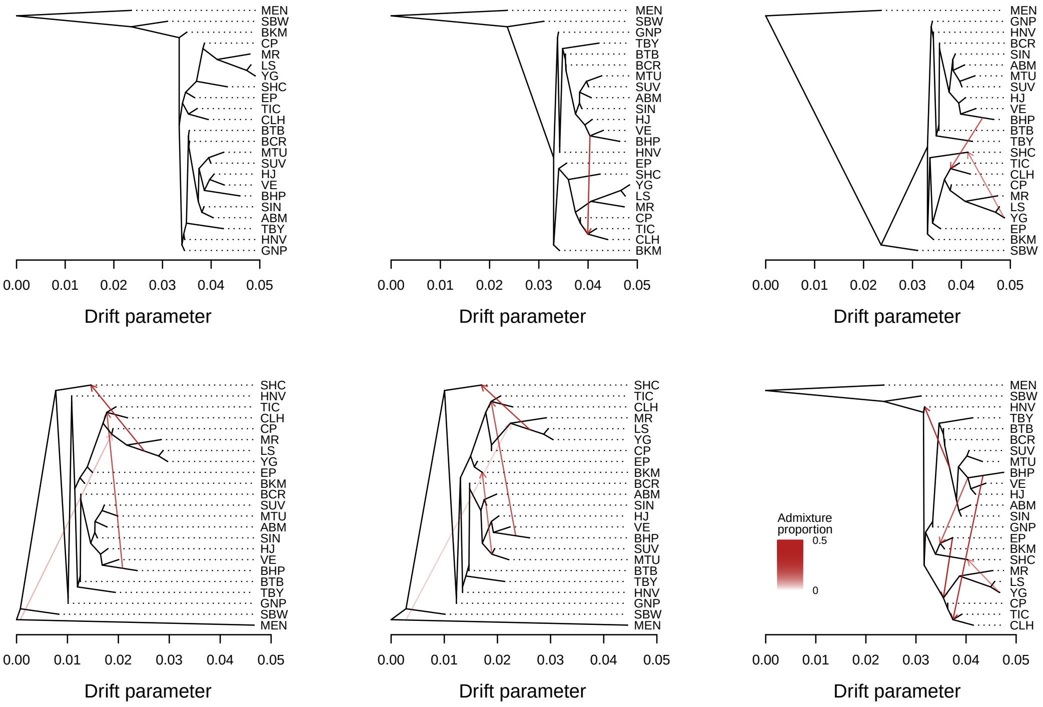

Figure S20: Population graphs for chromosome 19, with units in terms of a drift parameter proportional to evolutionary change and with the *L. argyrognomon* population (MEN) included as an outgroup. Graphs are shown for m = 0 (bifurcating tree) to five migration edges (red arrows). The admixture proportions associated with each are indicated by the intensity of each red arrow.

bioRxiv preprint doi: https://doi.org/10.1101/2025.11.28.691233; this version posted November 29, 2025. The copyright holder for this preprint (which was not certified by peer review) is the author/funder, who has granted bioRxiv a license to display the preprint in perpetuity. It is made available under aCC-BY 4.0 International license.

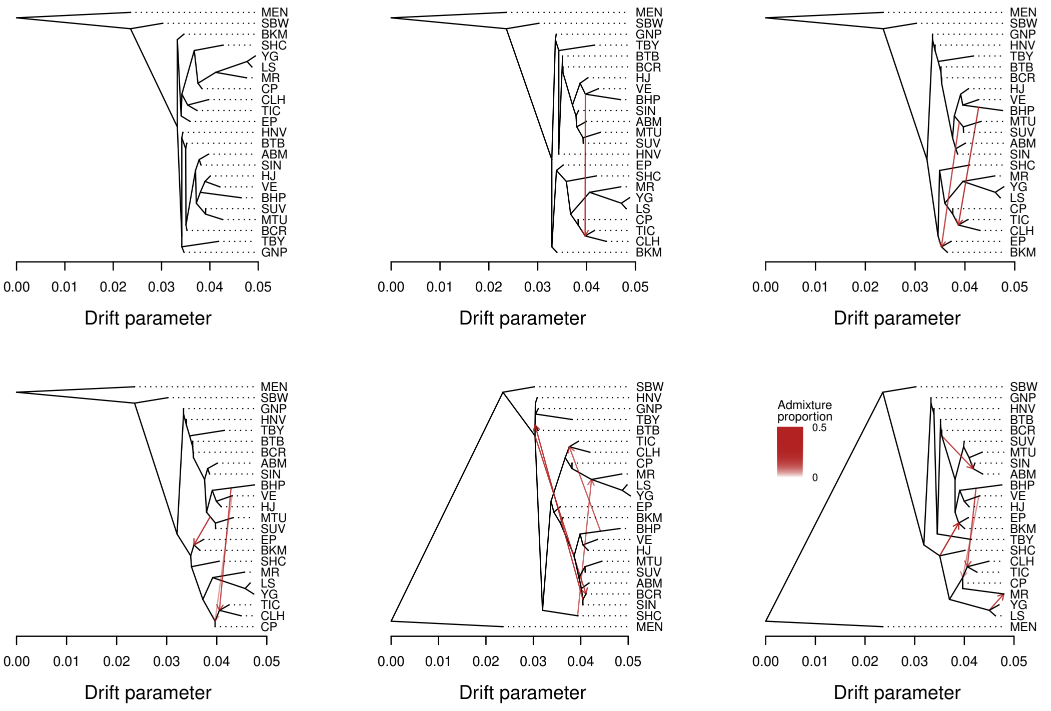

Figure S21: Population graphs for chromosome 20, with units in terms of a drift parameter proportional to evolutionary change and with the *L. argyrognomon* population (MEN) included as an outgroup. Graphs are shown for m = 0 (bifurcating tree) to five migration edges (red arrows). The admixture proportions associated with each are indicated by the intensity of each red arrow.

bioRxiv preprint doi: https://doi.org/10.1101/2025.11.28.691233; this version posted November 29, 2025. The copyright holder for this preprint (which was not certified by peer review) is the author/funder, who has granted bioRxiv a license to display the preprint in perpetuity. It is made available under aCC-BY 4.0 International license.

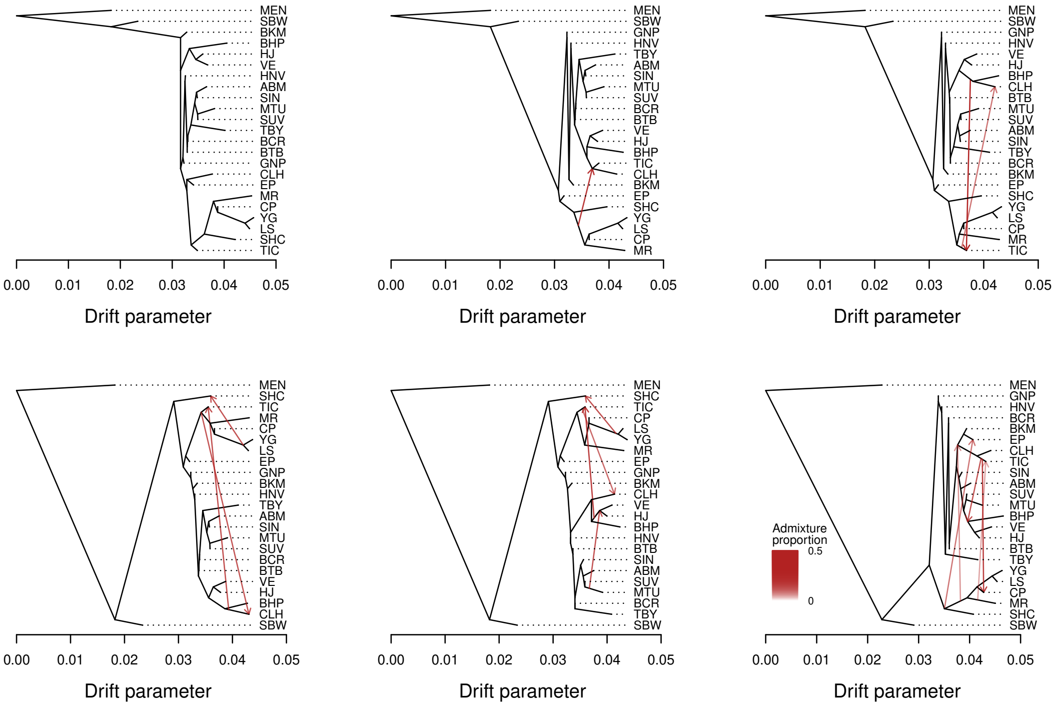

Figure S22: Population graphs for chromosome 21, with units in terms of a drift parameter proportional to evolutionary change and with the *L. argyrognomon* population (MEN) included as an outgroup. Graphs are shown for m = 0 (bifurcating tree) to five migration edges (red arrows). The admixture proportions associated with each are indicated by the intensity of each red arrow.

bioRxiv preprint doi: https://doi.org/10.1101/2025.11.28.691233; this version posted November 29, 2025. The copyright holder for this preprint (which was not certified by peer review) is the author/funder, who has granted bioRxiv a license to display the preprint in perpetuity. It is made available under aCC-BY 4.0 International license.

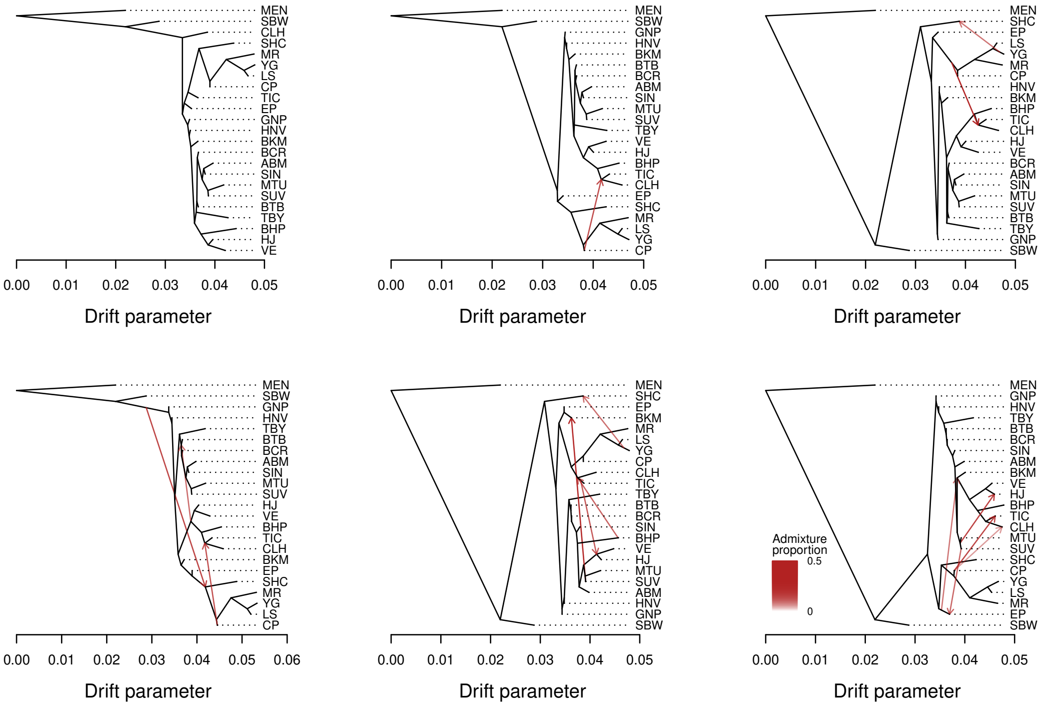

Figure S23: Population graphs for chromosome 22, with units in terms of a drift parameter proportional to evolutionary change and with the *L. argyrognomon* population (MEN) included as an outgroup. Graphs are shown for m = 0 (bifurcating tree) to five migration edges (red arrows). The admixture proportions associated with each are indicated by the intensity of each red arrow.

bioRxiv preprint doi: https://doi.org/10.1101/2025.11.28.691233; this version posted November 29, 2025. The copyright holder for this preprint (which was not certified by peer review) is the author/funder, who has granted bioRxiv a license to display the preprint in perpetuity. It is made available under aCC-BY 4.0 International license.

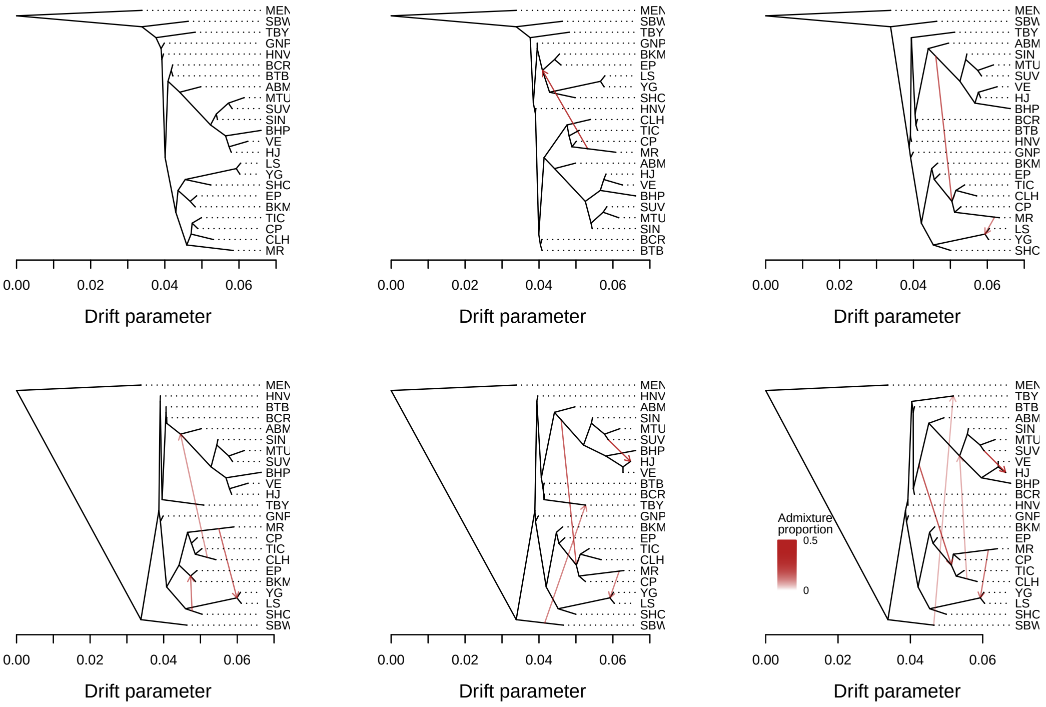

Figure S24: Population graphs for the Z chromosome, with units in terms of a drift parameter proportional to evolutionary change and with the *L. argyrognomon* population (MEN) included as an outgroup. Graphs are shown for m = 0 (bifurcating tree) to five migration edges (red arrows). The admixture proportions associated with each are indicated by the intensity of each red arrow.

bioRxiv preprint doi: https://doi.org/10.1101/2025.11.28.691233; this version posted November 29, 2025. The copyright holder for this preprint (which was not certified by peer review) is the author/funder, who has granted bioRxiv a license to display the preprint in perpetuity. It is made available under aCC-BY 4.0 International license.

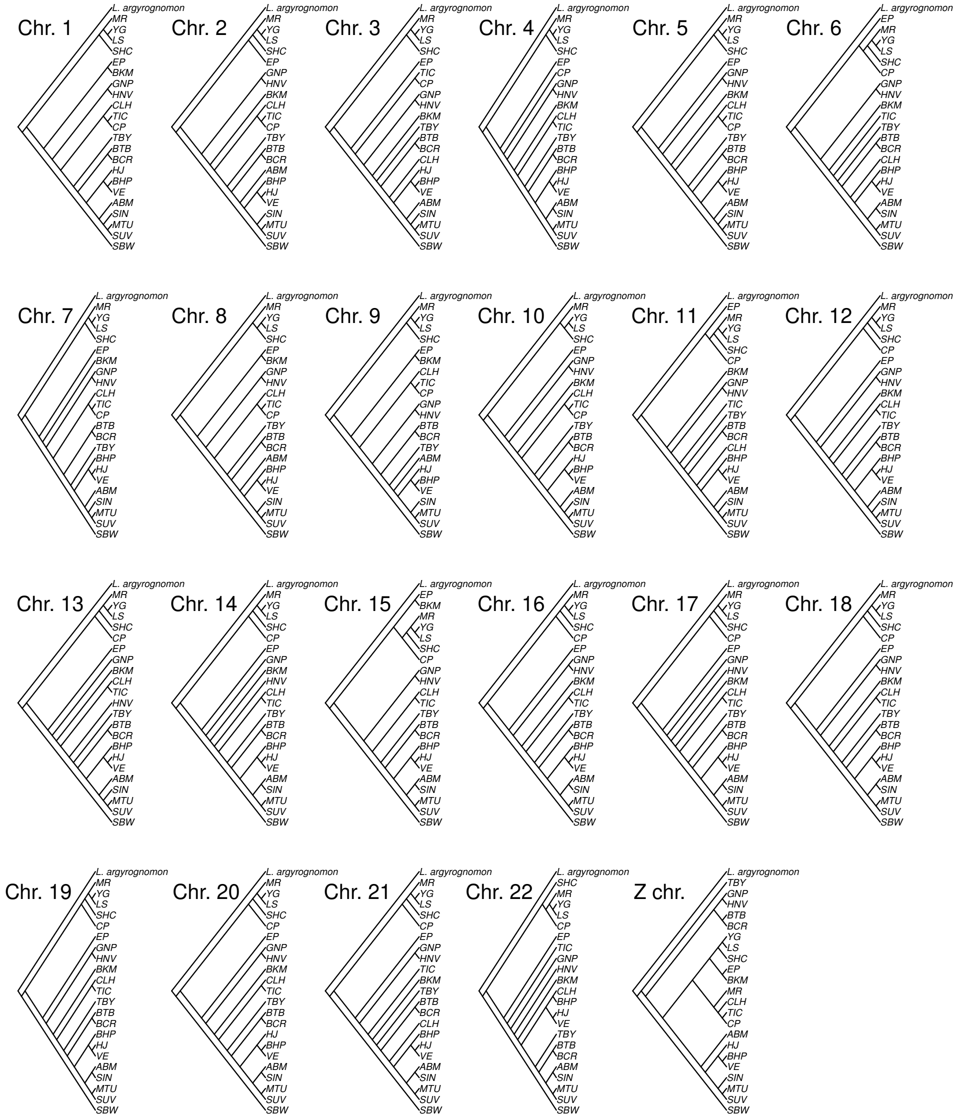

Figure S25: Tree topologies (as a cladogram) for each chromosome based on the `CASTER`-pair model. *Lycaeides argyrognomon* (population = MEN) was used as the outgroup.

bioRxiv preprint doi: https://doi.org/10.1101/2025.11.28.691233; this version posted November 29, 2025. The copyright holder for this preprint (which was not certified by peer review) is the author/funder, who has granted bioRxiv a license to display the preprint in perpetuity. It is made available under aCC-BY 4.0 International license.

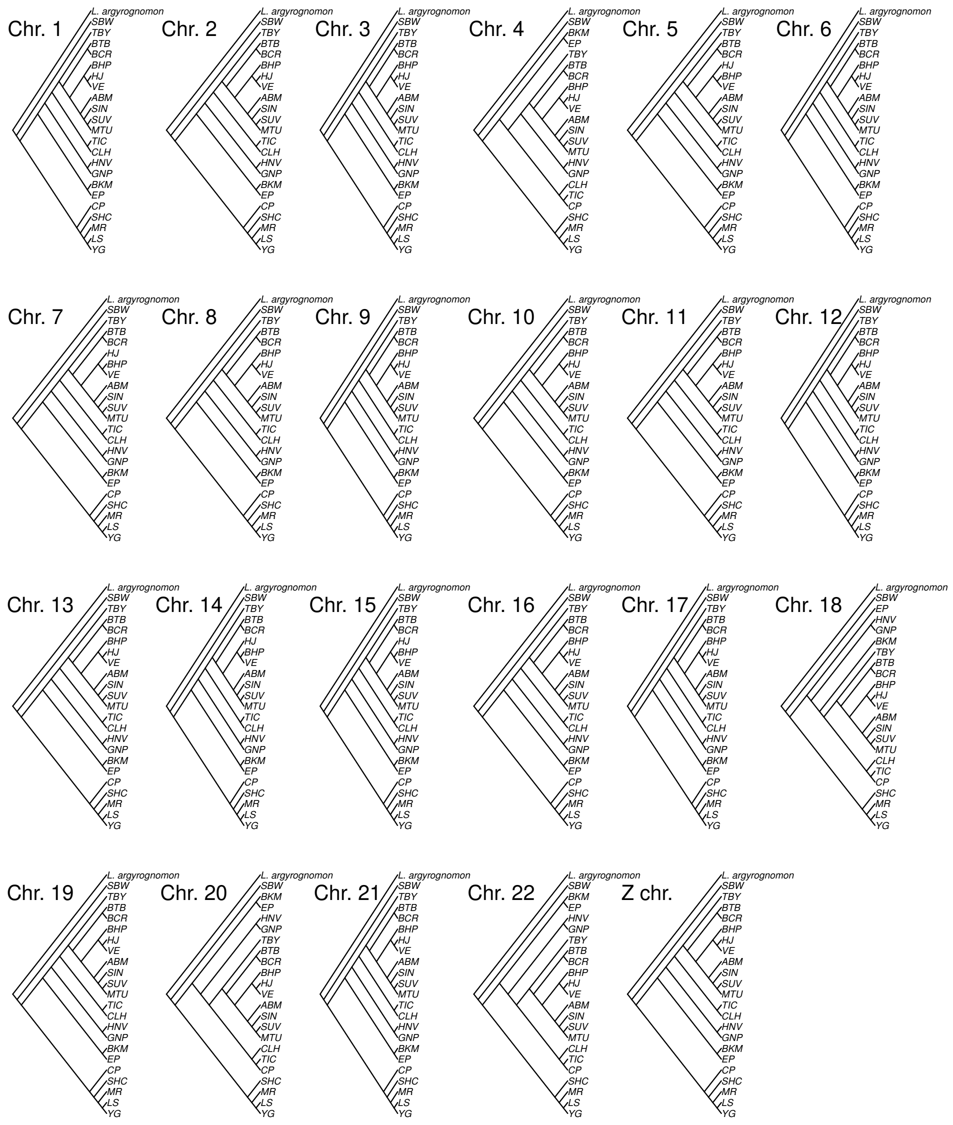

Figure S26: Example tree topologies (as a cladogram) based on the `CASTER`-site model with SNPs permuted among chromosomes. *Lycaeides argyrognomon* (population = MEN) was used as the outgroup. Results are shown for one permutation/randomization.

bioRxiv preprint doi: https://doi.org/10.1101/2025.11.28.691233; this version posted November 29, 2025. The copyright holder for this preprint (which was not certified by peer review) is the author/funder, who has granted bioRxiv a license to display the preprint in perpetuity. It is made available under aCC-BY 4.0 International license.

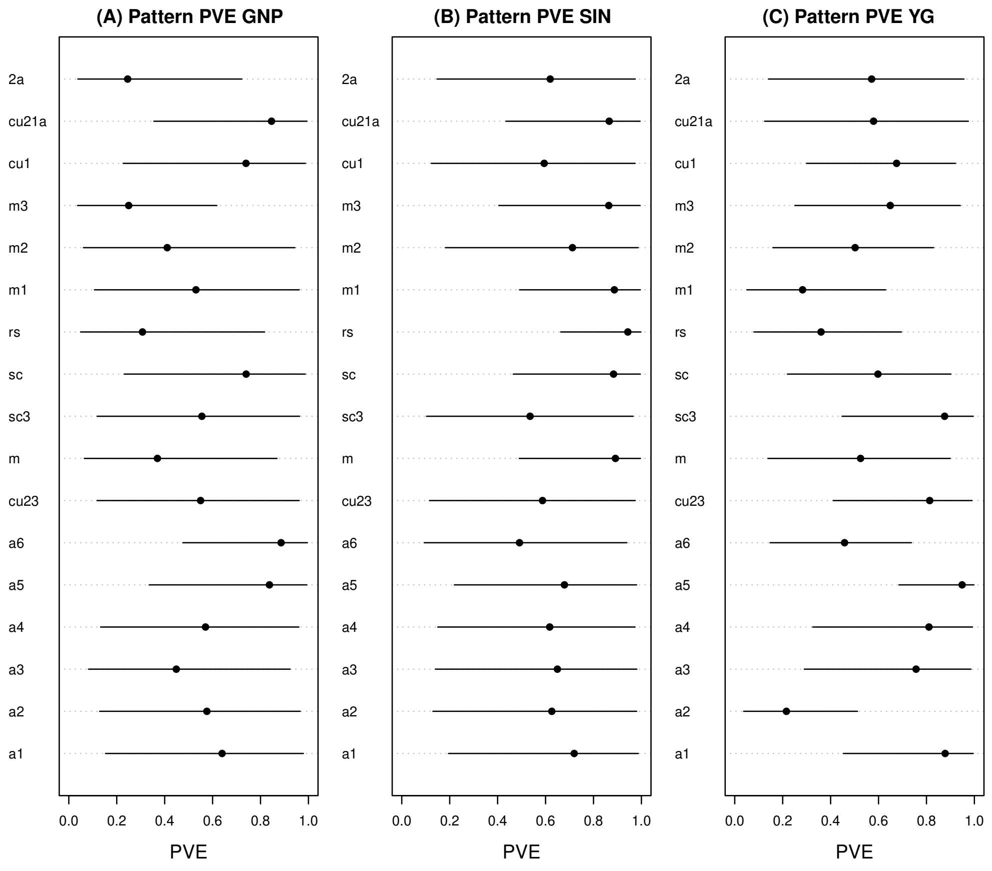

Figure S27: Dot plots depict Bayesian estimates of the proportion of wing pattern variation explained (PVE) by additive genetic effects for each of 17 pattern traits. Points and horizontal lines denote posterior medians and 80% equal-tail probability intervals, respectively. Results are shown for three populations: GNP (a), SIN (b) and YG (c). Pattern elements are defined in Figure 6.
